# Linking VOC assessment and cost-effectiveness for emission management in petroleum and petrochemical industrial estate

**DOI:** 10.1038/s41598-026-49628-3

**Published:** 2026-04-22

**Authors:** Peemapat Jookjantra, Jutarat Keawboonchu, Wissawa Malakan, Kiyoung Lee, Sarawut Thepanondh

**Affiliations:** 1 Department of Sanitary Engineering, Faculty of Public Health, Mahidol University, Bangkok, 10400 Thailand; 2 I-Grad, Faculty of Public Health, Mahidol University, Bangkok, 10400 Thailand; 3 Health Intervention and Technology Assessment Program Foundation (HITAP), Ministry of Public Health, Bangkok, Thailand; 4 Department of Environmental Health Sciences, Graduate School of Public Health, Seoul National University, Gwanak-gu, Seoul, 08826 Korea

**Keywords:** Volatile organic compounds (VOCs), Atmospheric dispersion modeling, Source apportionment, Emission inventory, Cost-effectiveness analysis, Emission management, Chemistry, Energy science and technology, Engineering, Environmental sciences

## Abstract

Managing volatile organic compound (VOC) emissions in industrial complexes requires clear links between emission sources, atmospheric dispersion, and their contributions to ambient exposure. This study links VOC assessment and cost-effectiveness analysis to support emission management in a petroleum and petrochemical industrial estate in Thailand. Annual emissions were estimated at 12,465 kg for benzene and 1,337 kg for 1,3-butadiene, with storage tanks dominating benzene releases (54.42%) and wastewater treatment systems governing 1,3-butadiene emissions (62.50%). Atmospheric dispersion modeling using AERMOD indicated compliance with Thai ambient air quality standards at all public-access receptors, with predicted concentrations below 1.7 µg/m³ for benzene and 0.33 µg/m³ for 1,3-butadiene, although elevated concentrations occurred near emission sources. Source apportionment revealed that storage infrastructure contributed 81–97% of ambient benzene concentrations at critical receptors, while wastewater treatment systems accounted for 74–76% of 1,3-butadiene exposure. Cost-effectiveness indicators and investment appraisal metrics were applied to evaluate emission control options. Lower-capital interventions were consistently prioritized. Activated carbon vapor recovery achieved a 95% reduction in benzene emissions, while hard-pipe wastewater modification reduced 1,3-butadiene emissions by 97.89% with minimal capital requirements. These findings indicate that substantial exposure reduction is achievable without reliance on capital-intensive controls.

**Supplementary Information:**

The online version contains supplementary material available at 10.1038/s41598-026-49628-3.

## Introduction

Rapid industrial development in emerging economies has intensified ambient air quality challenges, particularly within petroleum and petrochemical industrial complexes where volatile organic compounds (VOCs) are released from diverse operational activities^1,2^. VOCs play a central role in atmospheric chemistry by driving tropospheric ozone formation and secondary organic aerosol production, thereby influencing regional air quality and human exposure^3,4^. Among these compounds, benzene and 1,3-butadiene are of particular concern due to their classification as Group 1 human carcinogens and their regulation under national ambient air quality standards in many countries, including Thailand^5,6^. Long-term exposure to these hazardous VOCs poses elevated health risks for populations residing near industrial areas.

Global and regional inventories indicate that anthropogenic non-methane VOC emissions have increased substantially over recent decades, with industrial activities accounting for approximately 10–30% of total emissions worldwide^7–11^. In Southeast Asia, Thailand has emerged as a major petrochemical production hub, where large industrial estates are frequently located in close proximity to residential communities^12,13^. Source characterization studies conducted across petroleum refineries in China, Thailand, and other petroleum-producing regions consistently identify storage infrastructure as the dominant contributor to industrial VOC emissions, often exceeding 60% of total releases, followed by wastewater treatment systems and fugitive emissions from process equipment^14–16^. However, emission inventories alone do not account for atmospheric transport processes that determine ambient concentrations and population exposure, as the relationship between emission mass and receptor-level impact is modulated by source location, release characteristics, and meteorological conditions.

Atmospheric dispersion modeling provides a critical link between emission sources and ambient air quality impacts. Regulatory models such as AERMOD have been widely applied and validated for simulating VOC dispersion from petroleum and petrochemical facilities across diverse meteorological conditions^17–19^. In this study, AERMOD version 9.8.3 was applied as the regulatory Gaussian plume dispersion model, incorporating AERMET for meteorological preprocessing and AERMAP for terrain analysis, with emission inputs derived from source-specific measurement and modeling protocols covering six emission categories over the full calendar year 2022, and meteorological inputs processed through AERMET from a 100 m tower operated by the Thai Meteorological Department. By representing atmospheric transport and dispersion, AERMOD enables identification of spatial concentration patterns, assessment of compliance with air quality standards, and quantification of source-specific contributions to receptor exposure. When combined with receptor-based source apportionment techniques, dispersion modeling can reveal emission sources that disproportionately influence ambient concentrations despite relatively modest emission mass contributions.

While dispersion modeling and source apportionment can identify which emission sources most influence ambient concentrations, translating these findings into actionable management decisions additionally requires evaluation of the economic feasibility of alternative control strategies. Effective VOC emission management therefore requires control strategies that are both technically effective and economically feasible. Although mitigation measures such as leak detection and repair programs and vapor recovery systems can achieve capture efficiencies exceeding 60%^20–22^, economic evaluation of emission control options remains limited^23,24^. Financial appraisal tools such as net present value and internal rate of return are applied less frequently to industrial emission control prioritization, particularly in developing economies^25–27^. In this context, the limited integration of economic feasibility analysis with source-specific emission and exposure assessment represents a key methodological gap, particularly for industrial settings in developing economies where mitigation investment decisions require transparent prioritization tools. This study addresses this gap by integrating emission inventory, AERMOD atmospheric dispersion modeling, receptor-based source apportionment, and financial appraisal metrics (NPV and IRR) to support prioritization of VOC emission control in a petroleum and petrochemical industrial estate in eastern Thailand. The study focuses on benzene and 1,3-butadiene, two IARC Group 1 carcinogens regulated under Thai ambient air quality standards, and develops a transferable decision-support framework linking dominant emission sources, ambient concentration response, and economic feasibility of control alternatives.

## Methodology

### Study area

**Fig. 1:**
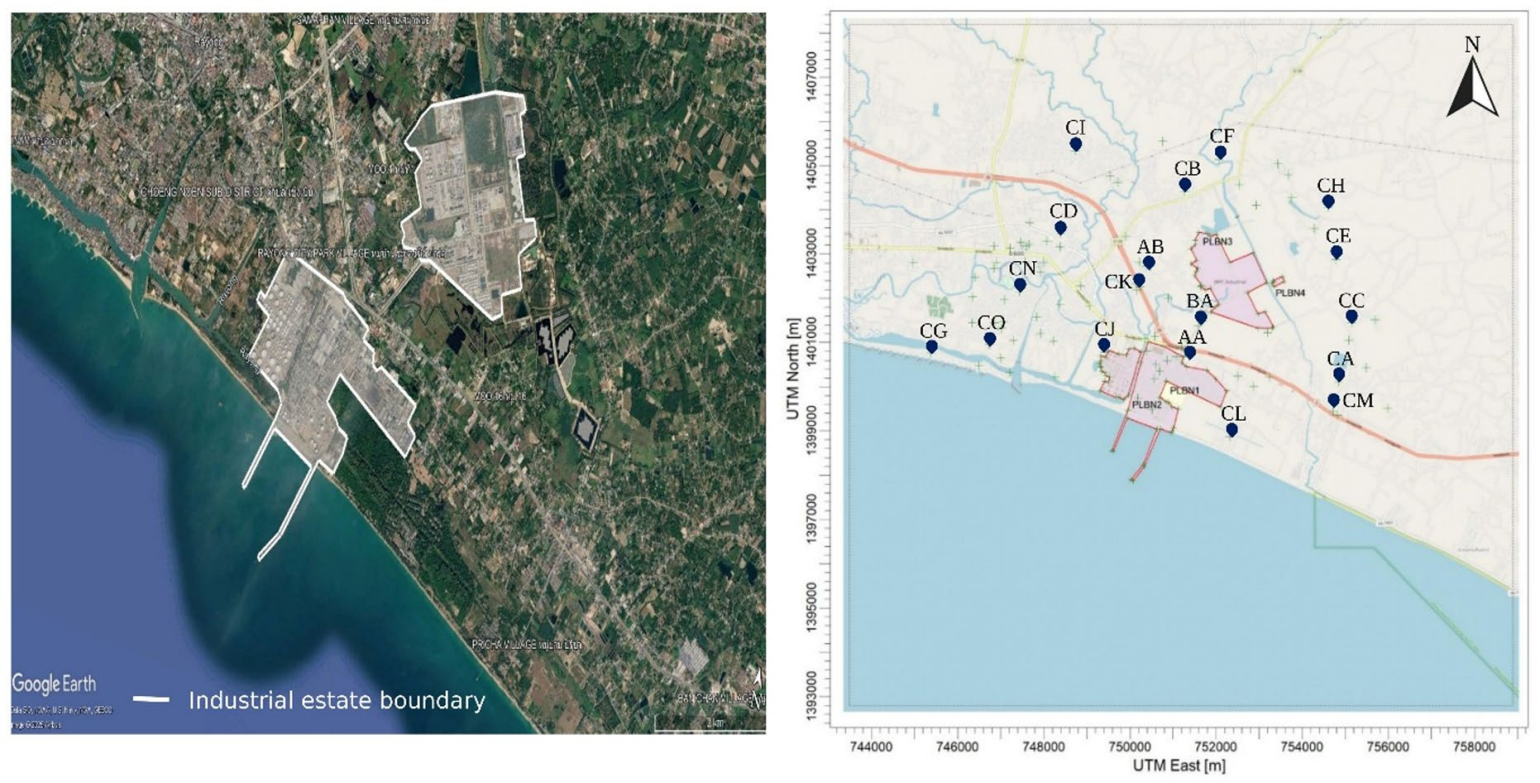
Study area and receptor locations surrounding the industrial estate in Rayong Province, Thailand. Left panel: satellite image from Google Earth Pro (Image © 2026 Airbus, https://earth.google.com); industrial estate boundary delineated by the authors. Right panel: generated using AERMOD version 9.8.3 (U.S. EPA, https://www.epa.gov/scram/air-quality-dispersion-modeling-preferred-and-recommended-models).

The study was conducted in a petroleum and petrochemical industrial estate located in Mueang Rayong District, Rayong Province, eastern Thailand (Fig. 1). The industrial estate covers an area of approximately 5,695 rai, with 63% allocated for industrial and warehouse purposes. Public utilities and infrastructure account for 16% of the estate, while green spaces and buffer zones cover 12%. Additionally, 4% of the area is designated for future development, and 4% is utilized for educational institutions. This industrial estate is centered at approximately 12.68°N, 101.28°E. This location is significant because it represents one of the largest integrated petroleum and petrochemical complexes in Southeast Asia, with multiple refining and petrochemical operations in close proximity to densely populated residential communities, creating a critical interface between industrial emissions and population exposure. The industrial complex is bisected by Sukhumvit Road into northern and southern operational zones and comprises multiple facilities. Benzene and 1,3-butadiene were selected as target compounds because they are classified as Group 1 human carcinogens by the International Agency for Research on Cancer (IARC)^5,6^, are regulated under Thailand’s National Ambient Air Quality Standards with annual average limits of 1.7 µg/m³ and 0.33 µg/m³ respectively, and are the two hazardous VOCs most directly associated with petroleum refining and petrochemical production processes in the study area. Major emission sources include six petroleum facilities (PTL1–PTL6), five petrochemical facilities (PTC1–PTC5), and a centralized wastewater treatment plant serving the estate. Eighteen discrete receptor locations were selected based on multiple criteria: (1) land use classification, prioritizing residential communities, educational institutions, healthcare facilities, religious sites, and government offices; (2) proximity to major emission sources within the industrial estate; (3) spatial distribution to capture both upwind and downwind conditions relative to prevailing wind directions; and (4) population density, focusing on areas with higher potential for human exposure. The receptor locations and their characteristics are detailed in Table S1. This selection ensures adequate spatial coverage of the populated areas surrounding the industrial estate while capturing the gradient of concentration decay with distance from emission sources.

Emission data and monitoring data were obtained from the petroleum and petrochemical industrial estate in Rayong Province, eastern Thailand. Facilities are identified using generic codes throughout this study to protect confidential business information under the data-sharing agreement between the research team and the industrial estate operator, consistent with standard practice in industrial emission studies.

### VOC emission inventory

VOC emissions were quantified for the period from 1 January to 31 December 2022 for six source categories, including combustion stacks, fugitive emissions from equipment components, flares, loading and unloading operations, storage tanks, and wastewater treatment units, representing a total of 142 emission points comprised 9 equipment component units (fugitive emissions), 8 flare system units, 42 combustion stacks, 81 storage tanks, 1 wastewater treatment plant (encompassing multiple unit operations), and 1 loading and unloading terminal unit, reflecting the operational complexity of the industrial estate and the relative scale of each source category. Emission estimates were developed using U.S. EPA-recommended protocols based on direct measurements and validated calculation models rather than generic emission factors, with source-specific methodologies and parameters summarized in Table 1. Operational data for emission estimation were obtained from facility records for the calendar year 2022 (1 January to 31 December). Stack emissions were quantified using continuous emission monitoring system (CEMS) data and periodic U.S. EPA Method 18 measurements. Flare emissions were estimated from flare gas flow meters and composition analyses. Fugitive emissions were quantified through annual leak detection and repair (LDAR) surveys using photoionization detectors following U.S. EPA Method 21, covering all accessible equipment components. Storage tank emissions were estimated using TANKS version 5.1 with facility-specific inputs including tank dimensions, roof configurations, stored material properties, throughput records, and meteorological data. Wastewater treatment emissions were modeled using WATER9 version 3.0 with influent VOC concentrations determined by purge-and-trap GC/MS analysis. Loading and unloading emissions were calculated from terminal throughput records, product vapor pressures, and transfer equipment specifications. All operational parameters, including flow rates, VOC concentrations, operating hours, chemical throughput, and control efficiencies, were collected under representative conditions spanning the full calendar year to capture seasonal variability in production volumes and meteorological conditions. Consistent with previous refinery and petrochemical source characterization studies, storage tanks, wastewater treatment systems, and equipment leaks were identified as dominant VOC emission sources, while cooling towers were excluded due to their negligible contribution relative to these categories^13,16^. Measurement uncertainty was estimated at approximately ± 20% for direct measurements conducted using U.S. EPA Method 18 and up to ± 50% for model-based estimates using TANKS and WATER9, in accordance with U.S. EPA guidance^34–36^.

The selection of six source categories is consistent with the U.S. EPA Emissions Estimation Protocol for Petroleum Refineries^36^, which identifies these categories as the principal VOC emission pathways requiring quantification in refinery-scale emission inventories. This categorization has been independently validated by multiple source characterization studies across diverse geographic and operational contexts. You et al^16^. confirmed that storage tanks, process vents, fugitive equipment leaks, and wastewater treatment are the dominant emission sources in Chinese petroleum refineries based on on-site measurements from multiple facilities. Jindamanee et al^13^. similarly reported that storage tanks, wastewater treatment, and combustion processes constitute the primary VOC release pathways from petroleum refineries in Thailand. Hini et al^10^. further demonstrated that these same categories account for the majority of VOC emissions in China’s petrochemical bases.

The comprehensive coverage of 142 individual emission points across all six categories, combined with source-specific quantification methods (Table 1), ensures that the emission inventory captures the dominant VOC release pathways within the industrial estate.

**Table 1:** Methods applied for VOC emission quantification by source category.

Source	Method	Parameter
Process fugitive	US.EPA Method 21^28^	Benzene 1,3-Butadiene
Flare	U.S. EPA AP-42 Chap. 13.5^29^	
Stack/combustion process	Direct measurement followed U.S. EPA Method 18^30^	
Storage tank	TANKS version 5.1^31^	
Wastewater treatment	WATER9 version 3.0^32^	
Loading/unloading at the terminal	U.S. EPA AP-42 Chap. 5.2^33^	

#### Fugitive emission estimation

Annual fugitive VOC emissions from process equipment were quantified using field screening measurements conducted during routine operations. Screening was performed with a photoionization detector equipped with a 10.6 eV krypton lamp (PID). Monitored components included valves, pumps, pressure relief devices, flanges, open-ended lines, sampling connections, and static mixers. Emission rates were calculated using the U.S. EPA correlation equation approach, with refinery- and chemical-manufacturing-specific parameters provided in Tables S2 and S3. Total organic compound emissions were estimated using Eq. (1).

1 $$\:{{\mathrm{e}}_{{\mathrm{TOC}}}}\:{\text{ = }}\:\sum {\:_{{\text{i = 1}}}^{\mathrm{n}}} \left\{ {\begin{array}{*{20}{c}} {{{\mathrm{e}}_{{\mathrm{0,i}}}}\left( {{\mathrm{0}}\:{\text{ = SV}} < {\mathrm{1}}} \right)} \\ {\:{\mathrm{a}} \cdot {\mathrm{SV}}_{\mathrm{i}}^{\mathrm{b}}\left( {{\mathrm{SV}}\:{\text{ = 100000}}} \right)} \\ {\:{{\mathrm{e}}_{{\mathrm{f,i}}}}\left( {{\text{1 = SV}} < {\mathrm{100000}}} \right)} \end{array}} \right.$$

where e_TOC_ is the total organic compound emission rate (kg/hr), SV is the background-corrected screening concentration at component i (ppmv isobutylene equivalent)^37^, e_0,i_ is the zero-reading baseline emission rate (kg/hr), *a* and *b* are empirically derived correlation parameters, and e_f, i_ represents the upper-bound emission rate for the screened component (kg/hr).

#### Flare emission estimation

VOC emissions from flare systems were estimated using a mass balance approach consistent with U.S. EPA AP-42 Chap. 13.5^29^. Although AP-42 does not present this approach as a formal equation, it is widely applied in regulatory permitting and atmospheric modeling studies to quantify unburned hydrocarbons released from industrial flares. Unburned VOC emissions were calculated using the following Eq. (2).

2 $$\:{\mathrm{VOC}}\:{\mathrm{Emission}}\:{\mathrm{Rate}}\:({\mathrm{kg/hr}})\:\:{\text{ = }}\:{{Q \times CVOC \times (1 - CE)}}$$

where Q is the volumetric flow rate of waste gas to the flare (m³/hr), C_VOC_ is the VOC concentration in the flared gas (kg/m³), and CE is the combustion efficiency. Facility-specific operational data were used to calculate average emission rates, which served as inputs for dispersion modeling and source contribution analyses.

#### Stack emission estimation

Stack emissions from combustion processes were quantified using U.S. EPA Method 18, which employs gas chromatographic analysis to determine VOC concentrations in exhaust streams^30^. These emissions originate primarily from natural gas and low-sulfur fuel oil combustion. Annual VOC mass emission rates were calculated following established formulations adapted for refinery and petrochemical applications^36^, as expressed in Eq. (3).

3 $$\:\mathrm{E}\mathrm{j}\mathrm{\:=\:}{\sum\:}_{\mathrm{j}}\mathrm{(}\mathrm{Q}\mathrm{j}{\times\:}\mathrm{C}\mathrm{j}{\times\:}\mathrm{h}\mathrm{j}\mathrm{)}$$

where j denotes individual VOC emission sources. The parameter E_j_ represents total annual VOC emissions from source j expressed in mg/year, while Q_j_ indicates the volumetric flow rate (m^3^/hr) of emissions. The concentration of VOCs at source j is denoted by C_j_ (mg/m^3^), and h_j_ signifies the operational hours per year (hr/year) for each emission source.

#### Storage tank emission estimation

VOC emissions from aboveground storage tanks were quantified using TANKS version 5.1, a cloud-based model released by the U.S. EPA in October 2024^31^. The model applies AP-42 Chap. 7.1 methodologies to estimate standing and working losses associated with temperature fluctuations, barometric pressure changes, and material throughput^38,39^. Model inputs included site-specific meteorological conditions, tank design and construction parameters, operational data, chemical composition of stored materials, and vapor control characteristics.

#### Wastewater treatment emission estimation

Atmospheric VOC emissions from wastewater treatment processes were estimated using WATER9 version 3.0, a U.S. EPA mass balance model incorporating physicochemical properties and biodegradation kinetics^32^. The model accounts for phase partitioning, volatilization, and biological removal across sequential treatment units, providing compound-specific emission estimates suitable for regulatory and air quality impact assessments^40,41^.

#### Loading and unloading emission estimation

VOC emissions from product loading and unloading operations were estimated using U.S. EPA AP-42 Sect. 5.2 methodologies. Emissions arise from vapor displacement during loading, breathing losses during transport, and ballasting operations in marine systems^33^. Emission factors accounting for vapor saturation, true vapor pressure, molecular weight, temperature, and control efficiency were applied using Eq. (4).

4 $$\:{\mathrm{L}}_{\mathrm{L}}\mathrm{=12.46}\frac{\mathrm{SPM}}{\mathrm{T}}\left(\mathrm{1-}\frac{\mathrm{eff}}{\mathrm{100}}\right)$$

where L_L_ is the VOC emission mass per unit volume of transferred product (kg/m³), S is the vapor saturation coefficient (Table S4), P is the equilibrium vapor pressure at operating conditions (kPa), M is the vapor molecular mass (g/mol), and T is the bulk liquid temperature during transfer (K).

### Atmospheric dispersion modeling

#### AERMOD model description

Atmospheric dispersion was simulated using AERMOD version 9.8.3, a steady-state Gaussian plume model approved by U.S. EPA and Thai regulatory authorities for near-field applications within 50 km^42,43^. The modeling system incorporated AERMET for meteorological preprocessing and AERMAP for terrain analysis. Benzene was treated as inert due to its long atmospheric lifetime of approximately nine days^3^. Although 1,3-butadiene exhibits a shorter lifetime (1–9 h)^44^, near-source transport dominates within the modeled domain, justifying the non-reactive assumption.

#### Model configuration and meteorology

The modeling domain covered 7 × 7 km area, centered at UTM coordinates X = 751194.69 m and Y = 1400488.96 m, with a uniform receptor grid resolution of 250 m. Model inputs included the 2022 emission inventory, SRTM-based terrain data processed using AERMAP, and annual surface and upper-air meteorological observations processed through AERMET. Emission sources comprised six categories as detailed in Tables S5–S10.

Terrain elevations ranged from 1 to 402 m above sea level, with most of the area below 10 m, supporting the application of flat-terrain assumptions. Meteorological data were obtained from a 100 m tower operated by the Thai Meteorological Department. Receptor placement excluded restricted industrial zones in accordance with ambient air quality regulations^45^, focusing on surrounding communities and public-access areas. Model configuration details are summarized in Table 2.

**Table 2:** AERMOD modeling system^42,43^ specifications.

Parameter	Configuration Details
Computational domain	- Origin coordinates (UTM): X = 751194.69 m, Y = 1400488.96 m - No. of X and Y Axis Receptors: 68 - Grid resolution: 250 m intervals (both axes) - Length of X and Y (m): 17,000
Emission inventory	Benzene and 1,3-butadiene emission database (2022)
Meteorology inputs	AERMET-processed surface and upper air observations (2022)
Topographic data	SRTM digital elevation model (SRTM3/SRTM1 resolution)
Model outputs	Annual-averaged pollutant concentrations

### Decision-support analysis

This section presents a decision-support framework for prioritizing VOC emission control strategies in the petroleum and petrochemical industrial estate. The analysis integrates management scenarios, cost-effectiveness indicators, and investment appraisal metrics to support source-oriented air quality management focusing on benzene and 1,3-butadiene.

#### Management scenarios and control measures

The decision-support analysis was conducted under three management scenarios representing different emission control conditions within the industrial estate (Table S11). Scenario 1 represents baseline conditions without emission controls, while Scenario 2 (business-as-usual; BAU) reflects current operations with existing control technologies and measured emissions and served as the reference case. Scenario 3 evaluates four proposed emission control measures for each pollutant, selected based on dominant source categories identified through source contribution analysis. The proposed measures include equipment upgrades and system modifications targeting major VOC emission sources, with control efficiencies, capital investment requirements, and expected emission reductions defined based on industrial practice and historical installation data. These scenarios formed the basis for subsequent cost-effectiveness and feasibility assessments.

#### Cost-effectiveness indicators

Two complementary metrics were used to prioritize emission control options: cost per unit VOC emission reduction (THB per kg VOC reduced) and cost per unit reduction in ambient VOC concentration (THB per 0.1 µg/m³ reduced). Emission reductions were quantified relative to the business-as-usual scenario, and indicator values were derived from capital investment costs for each control option. The concentration-based metric directly links emission control performance to receptor-level air quality outcomes, supporting source-oriented air quality management decisions.

#### Investment appraisal metrics (NPV and IRR)

Net present value (NPV) and internal rate of return (IRR) were applied to evaluate the financial feasibility of prioritized emission control options as supplementary decision-support tools. NPV quantifies the difference between discounted cost savings and capital investment costs over the project lifetime, accounting for the time value of money and investment uncertainty^46^^[,47^, and was calculated using Eq. (5).

5 $$\:\mathrm{NPV\:=\:-}{\text{}\mathrm{C}}_{\mathrm{0}}\mathrm{+}\sum\:_{\mathrm{t=1}}^{\mathrm{n}}\frac{\mathrm{Rt}}{{\mathrm{(1+r)}}^{\mathrm{t}}}\text{}\text{}$$

where R_t_ represents annual cost savings relative to baseline conditions, C_0_ is the initial capital investment, r is the discount rate, and t denotes the time period. Positive NPV values indicate economic feasibility, although NPV alone may be insufficient for comparing projects with substantially different capital requirements^48^.

To complement NPV, IRR was calculated as the discount rate at which the net present value equals zero, following Eq. (6).

6 $$\:\mathrm{NPV\:=\:0\:=\:-}{\text{}\mathrm{C}}_{\mathrm{0}}\mathrm{+}\sum\:_{\mathrm{t=1}}^{\mathrm{n}}\frac{\mathrm{R}\mathrm{t}}{{\mathrm{(1+IRR)}}^{\mathrm{t}}}\text{}\text{}\text{}$$

Projects with IRR values exceeding the opportunity cost of capital were considered feasible, whereas options with IRR below this threshold were deemed less suitable for implementation^48^^[,49^.

Capital investment costs were estimated based on historical project data from similar emission control installations at comparable facilities (2020–2022). Historical costs were adjusted to 2022 Thai Baht to account for inflation and cost escalation, following standard chemical engineering cost estimation practice. Cost estimates include equipment procurement, installation labor, engineering, and 15% contingency. The estimation methodology follows established chemical engineering practices^46^^[,50^. A discount rate of 10% was applied, consistent with petrochemical industry benchmarks and international practice in developing economies^46^^[,51,52^^[,53^. Equipment lifetimes followed industry standards, with a 20-year project period adopted in line with petrochemical depreciation schedules^50^^[,54^^[,55^. Only capital costs were considered, as operating and maintenance (O&M) costs were minimal or highly variable across facilities. This capital-cost-only approach was adopted for three reasons. First, the economic analysis is intended as a comparative ranking tool rather than a definitive project-level financial appraisal, consistent with established investment screening frameworks applied to industrial emission control^55^^[,56^^[,57^. Second, O&M cost data for emission control systems in Thai petrochemical facilities are not systematically documented, and available estimates from international sources may not reflect local labor, energy, and material costs. Third, capital cost comparison is widely accepted in early-stage industrial decision-making to identify preferred investment options prior to detailed engineering cost studies^46^^[,50^.

### Quality assurance and quality control

To ensure data reliability and reproducibility, standardized quality control procedures were implemented across all measurement and modeling components. Stack VOC samples were collected in 1-L Tedlar bags and analyzed using U.S. EPA Method 18, achieving relative standard deviations below 30%, correlation coefficients greater than 0.95, and detection limits of 0.05 µg/m³ for benzene, toluene, and xylene^30^. Fugitive VOCs were measured using a photoionization detector calibrated annually with NIST-traceable standards (± 3% accuracy; Certificate No. RA 052/25) following U.S. EPA Method 21, incorporating hourly background correction, replicate measurements, and quality control checks that yielded relative standard deviations below 15%^28^. Wastewater VOC concentrations were determined by purge-and-trap GC/MS with analytical precision within 10% and detection limits below 2.0 µg/m³. Flare emissions were estimated assuming combustion efficiencies of 98–99.5%^29^, while emissions from loading and unloading operations were calculated using U.S. EPA AP-42 Chap. 5.2 with associated uncertainties of ± 30–50%^33^. Model-based estimates from TANKS version 5.1 were verified through consistency checks of meteorological inputs and material throughput, with overall uncertainties estimated at approximately ± 20% for direct measurements and ± 50% for model-based calculations, consistent with U.S. EPA uncertainty frameworks. All modeled concentrations are reported to two significant figures, consistent with the precision of the emission estimates and modeling uncertainty ranges. Emission values are reported as integers for totals exceeding 100 kg and to one decimal place for smaller source-specific contributions, consistent with the ± 20–50% uncertainty range of the estimation methods.

## Results

### Emission inventory in the BAU scenario

**Fig. 2:**
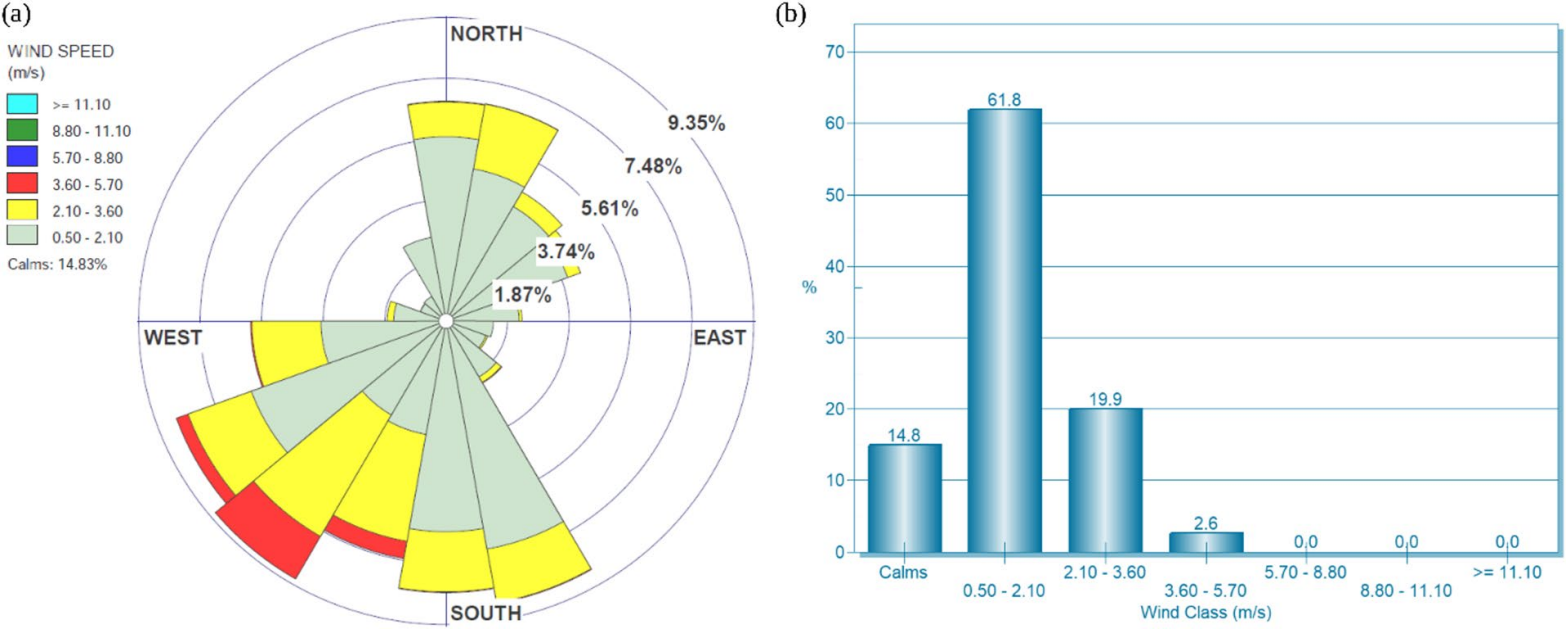
(**a**) Wind rose plot of study area and (**b**) % distribution of wind classes of Rayong Province, Thailand in 2022.

Under the business-as-usual (BAU) scenario, total benzene emissions in 2022 were estimated at 12,465 kg across the petroleum and petrochemical industrial estate (Table 3). Storage tanks were the dominant source, contributing 6,784 kg (54.42%), followed by combustion stacks (3,815 kg; 30.60%) and wastewater treatment units (1,047 kg; 8.40%), together accounting for approximately 93% of total emissions (Fig. 2b). This distribution is consistent with previous refinery and petrochemical studies identifying storage-related emissions as the primary contributor to facility-wide VOC releases^10^^[,13^^[,14^. Minor sources, including flares, loading and unloading operations, and fugitive emissions, collectively contributed less than 7%. At the facility level (Fig. 2a), storage tanks at PTL1 dominated benzene emissions (47.48%), followed by combustion stacks at PTC1 (30.36%). Emissions were highly concentrated within the storage category, with a single fixed-roof tank (69T088A) accounting for 79.26% of storage-related emissions (Table S12), consistent with previous observations highlighting storage infrastructure and combustion units as major benzene emitters^14^^[,58^^[,59^.

Total emissions of 1,3-butadiene under BAU conditions were estimated at 1,337 kg in 2022 (Table 3). In contrast to benzene, wastewater treatment units were the dominant source, contributing 836 kg (62.50%) of total releases (Fig. 2d), followed by loading and unloading operations (304 kg; 22.76%) and storage tanks (98 kg; 7.33%), with the remaining sources collectively accounting for less than 1%. This dominance reflects the physicochemical properties of 1,3-butadiene, a boiling point of − 4.5 °C, vapor pressure of approximately 2,110 mmHg at 25 °C, and Henry’s law constant of approximately 7.6 × 10⁻² atm·m³/mol at 25 °C^60^, which make it highly susceptible to volatilization from aqueous surfaces exposed to air through open sumps, drop pipes, and aeration basins. Source-level analysis (Fig. 2c) revealed that emissions were highly centralized, with waste drop pipe 2 alone contributing approximately 98% of total wastewater-related releases (Table S13), reflecting intense localized volatilization driven by direct wastewater-air contact without vapor containment. Conversely, storage tanks contributed minimally because only three tanks at PTC3 stored PBDE latex containing 1,3-butadiene (Table S12), and their vertical fixed-roof configurations limited evaporative losses. Flare systems and combustion stacks were also negligible due to high combustion efficiencies (98–99.5%) that effectively destroy this compound under normal operating conditions^61^. This emission pattern is consistent with prior studies identifying wastewater treatment as a dominant release pathway for 1,3-butadiene in petrochemical operations^61^^[,62^.

Overall, the BAU emission inventory demonstrates pollutant-specific source dominance, indicating that effective emission management requires differentiated control strategies targeting storage tanks for benzene and wastewater treatment systems for 1,3-butadiene.

**Table 3:** Emission inventory of benzene and 1,3-butadiene.

Sources	Plant	Benzene (kg/year)	Total benzene (kg/year)	1,3-Butadiene (kg/year)	Total 1,3-butadiene emission
Fugitive	PTC1	125	166	-	99.2
	PTC2	31.3		-	
	PTC3	-		74.3	
	PTC4	-		22.3	
	PTC5	7.1		2.5	
	PTL1	0.5		-	
	PTL2	0.7		-	
	PTL3	0.6		-	
	PTL5	1.0		-	
Flares	PTC1	44.9	106	-	-
	PTC2	< 0.1		-	
	PTC4	60.5		-	
	PTC5	0.2		-	
	PTL1	0.1		-	
	PTL2	< 0.1		-	
	PTL4	0.1		-	
	PTL5	0.3		-	
Stacks	PTC1	3,784	3,814	-	-
	PTC2	0.5		-	
	PTC4	12.5		-	
	PTC5	1.0		-	
	PTL1	2.8		-	
	PTL2	3.8		-	
	PTL4	2.1		-	
	PTL5	7.4		-	
Storage tanks	PTC1	92.2	6,784	-	98
	PTC2	374		-	
	PTC3	-		98	
	PTC4	101		-	
	PTC5	69.1		-	
	PTL1	5,967		-	
	PTL2	77.7		-	
	PTL5	37.1		-	
	PTL6	65.7		-	
Wastewater treatment	WWTP	1,047	1,047	836	836
Loading & unloading	PTL6	548	548	304	304
**Total emission**			**12**,**465**		**1**,**337**

### Spatial distribution and modeled VOC concentrations

Table 4 presents the modeled annual mean concentrations of benzene and 1,3-butadiene at individual receptor locations in 2022. Benzene concentrations ranged from 0.005 to 0.58 µg/m³, while 1,3-butadiene ranged from < 0.001 to 0.020 µg/m³. All predicted concentrations were well below the Thai annual ambient air quality standards of 1.7 µg/m³ for benzene and 0.33 µg/m³ for 1,3-butadiene, indicating regulatory compliance across the study area.

Notable spatial variability was observed among receptors. Receptor CJ exhibited the highest annual benzene concentration (0.58 µg/m³), with source apportionment indicating that chemical storage tanks accounted for 0.56 µg/m³ of this value (Table 4). This finding highlights the dominant influence of storage tank emissions on localized benzene levels near industrial operations, consistent with previous studies^63^^64^,. In contrast, most receptors recorded substantially lower benzene concentrations (≤ 0.071 µg/m³), primarily associated with evaporative releases from storage and handling activities.

**Fig. 3:**
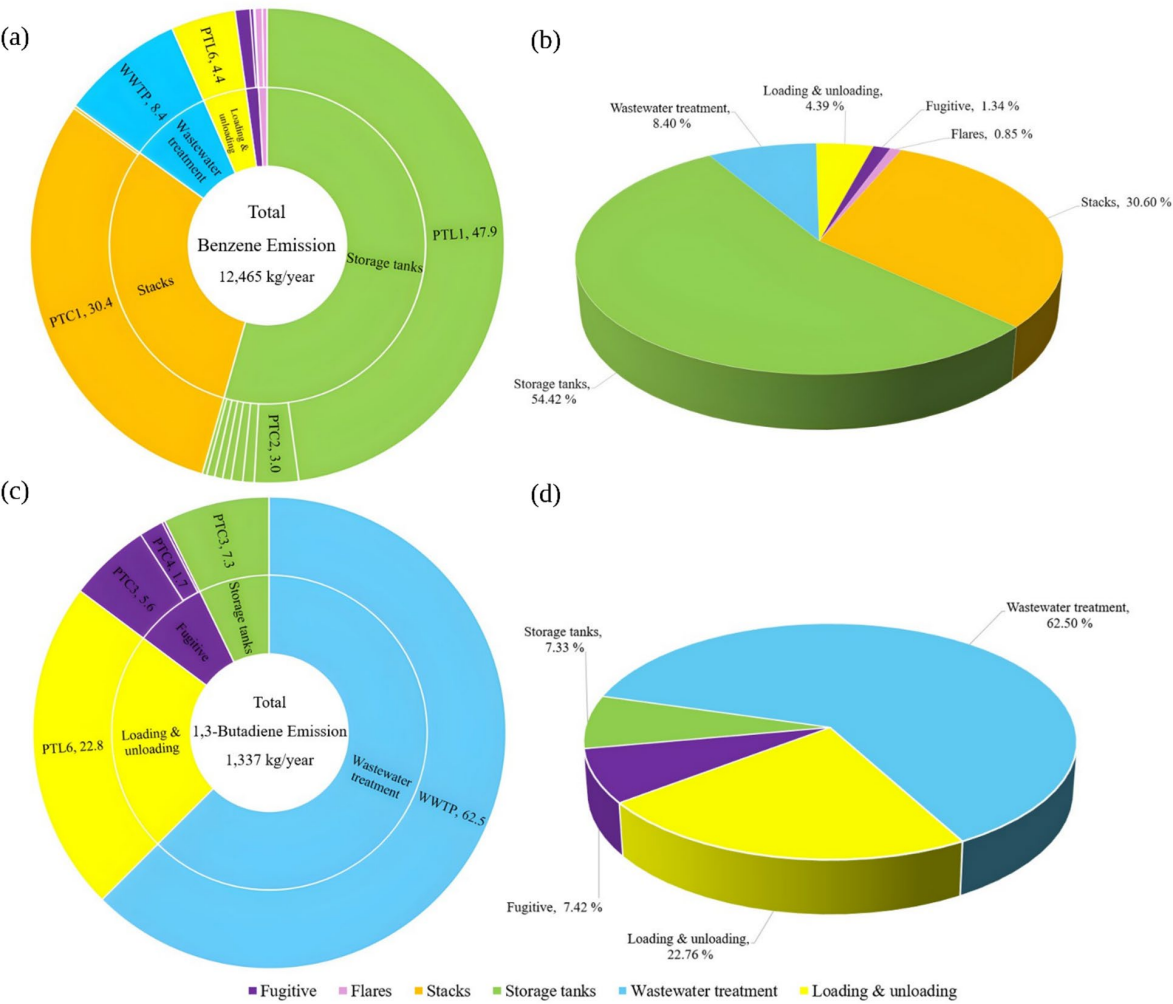
Emission inventory of (**a**) benzene and (**c**) 1,3-butadiene and percentage contributions of (**b**) benzene and (**d**) 1,3-butadiene in the petroleum and petrochemical industrial estate.

**Fig. 4:**
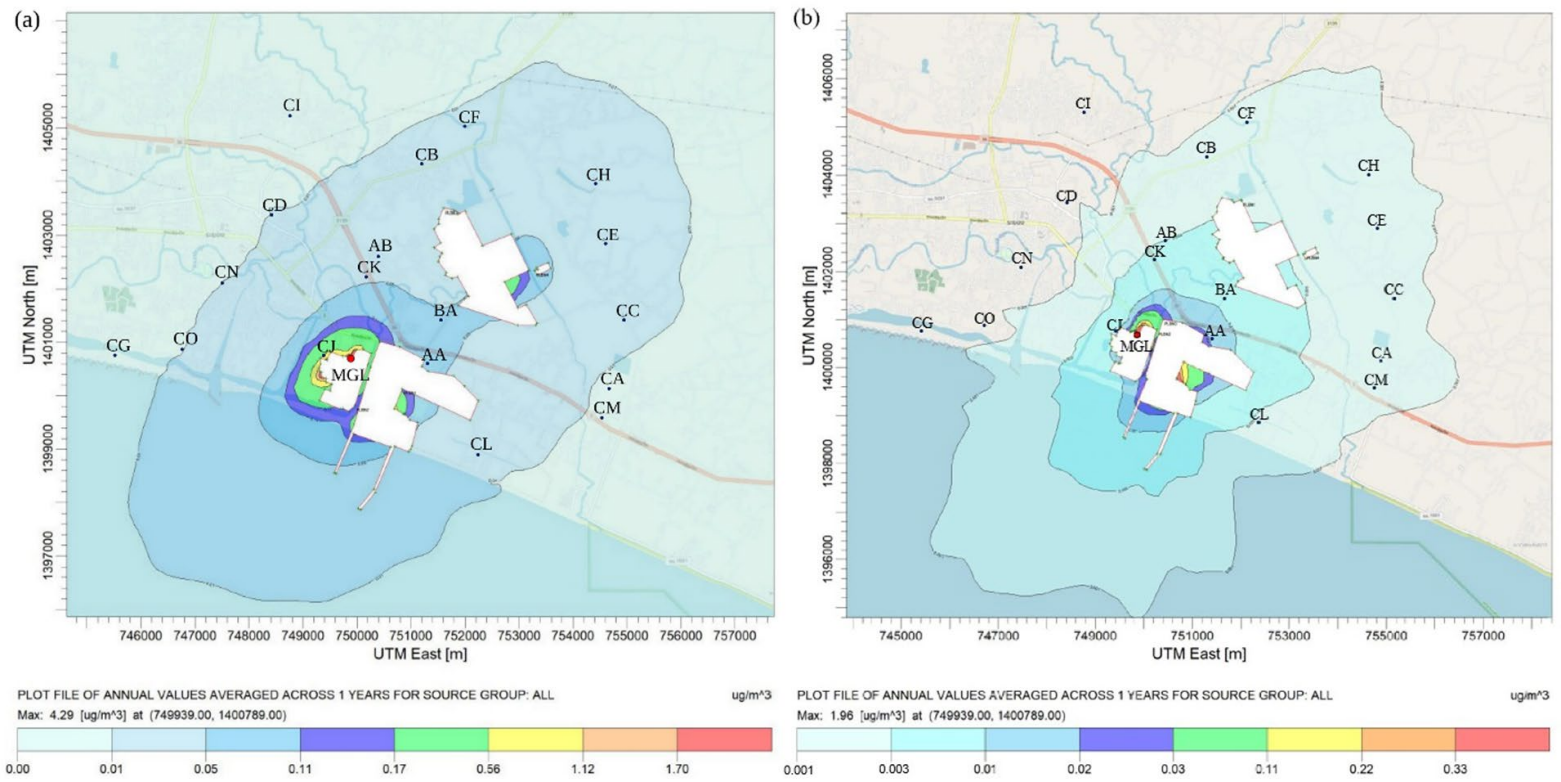
Spatial distribution of predicted annual concentration of (**a**) benzene and (**b**) 1,3-butadiene. Generated using AERMOD version 9.8.3 (U.S. EPA, https://www.epa.gov/scram/air-quality-dispersion-modeling-preferred-and-recommended-models).

The spatial distributions of benzene and 1,3-butadiene are illustrated in Fig. 3. For benzene (Fig. 3a), the maximum modeled annual average concentration (MGL) reached 4.29 µg/m³ at coordinates (UTM Zone 47 N, WGS84) 749939.00 m E and 1400789.00 m N, located within the industrial estate near chemical storage facilities. This hotspot coincided with receptor CJ, reinforcing the role of storage tanks as dominant sources. A similar pattern was observed for 1,3-butadiene (Fig. 3b), with the highest modeled concentration (1.96 µg/m³) occurring within the estate. Receptor AA recorded the highest receptor-level concentration (0.020 µg/m³), with slightly elevated levels also observed at receptors BA, AB, and CJ, reflecting proximity to emission sources. Concentrations at receptors farther from the industrial area were near or below detection limits, consistent with previous reports of elevated VOC levels in industrially influenced areas^65^^66^,. Model verification was conducted using multiple lines of evidence, following the approach recommended by U.S. EPA Model Clearinghouse guidelines^67^. First, the spatial concentration patterns produced by AERMOD were consistent with the prevailing westerly to southwesterly wind climatology observed in the study area (Fig. 4), with lower concentrations at upwind locations such as CG and CI, and higher values at receptors positioned near major emission sources or in downwind communities. The highest benzene concentration at receptor CJ (0.58 µg/m³) was attributable to its immediate proximity to dominant storage tank sources rather than wind transport alone, as confirmed by source apportionment analysis (Table 4). Second, maximum ground-level concentrations occurred within expected distances from major emission sources (approximately 0.5–1.0 km), consistent with Gaussian dispersion behavior for near-field applications. Third, predicted annual mean concentrations at ambient receptors (maximum 0.58 µg/m³ for benzene and 0.020 µg/m³ for 1,3-butadiene) were within the range of concentrations reported by the Pollution Control Department for Rayong Province monitoring stations^68^, where benzene levels have been documented to frequently approach or exceed the national standard of 1.7 µg/m³. Fourth, source contribution rankings (storage tanks dominating benzene, wastewater treatment dominating 1,3-butadiene) were consistent with previously published studies conducted in comparable petrochemical industrial areas in Thailand^12^^[,61^^[,63^. Fifth, the AERMOD modeling system has undergone extensive regulatory validation by U.S. EPA for facility-scale applications^43^, and its performance characteristics are well-documented for the source types and spatial scales examined in this study. While these verification approaches do not constitute a formal statistical model evaluation, they collectively support confidence in the predicted spatial patterns, source attribution, and relative concentration magnitudes reported in this study.

**Table 4:** Modeled annual average concentrations of benzene and 1,3-butadiene at receptors.

Code receptors	Annual concentrations of benzene (µg/m^3^)						
	Stacks	Flares	Fugitive	Storage tanks	Loading/ unloading	Wastewater treatment	All
AA	0.009	< 0.001	0.001	0.039	0.006	0.016	0.071
AB	0.002	< 0.001	< 0.001	0.023	0.002	0.001	0.029
BA	0.016	< 0.001	0.002	0.027	0.004	0.007	0.055
CA	0.001	< 0.001	< 0.001	0.005	< 0.001	0.002	0.009
CB	0.002	< 0.001	< 0.001	0.009	0.001	0.001	0.013
CC	0.004	< 0.001	< 0.001	0.007	0.001	0.001	0.013
CD	0.001	< 0.001	< 0.001	0.005	< 0.001	< 0.001	0.007
CE	0.010	< 0.001	0.001	0.007	0.001	0.001	0.019
CF	0.002	< 0.001	< 0.001	0.007	0.001	0.001	0.011
CG	0.001	< 0.001	< 0.001	0.005	< 0.001	< 0.001	0.006
CH	0.008	< 0.001	< 0.001	0.006	0.001	0.001	0.017
CI	0.001	< 0.001	< 0.001	0.004	< 0.001	< 0.001	0.005
CJ	0.002	< 0.001	< 0.001	0.56	0.014	0.002	0.58
CK	0.002	< 0.001	< 0.001	0.032	0.003	0.002	0.039
CL	0.001	< 0.001	< 0.001	0.009	0.001	0.003	0.014
CM	0.001	< 0.001	< 0.001	0.005	< 0.001	0.002	0.008
CN	0.001	< 0.001	< 0.001	0.008	< 0.001	< 0.001	0.010
CO	0.001	< 0.001	< 0.001	0.008	0.001	< 0.001	0.009
Annual concentrations of 1,3-butadiene (µg/m^3^)							
AA	-	-	0.002	0.002	0.003	0.012	0.020
AB	-	-	< 0.001	< 0.001	0.001	0.001	0.003
BA	-	-	0.001	0.001	0.002	0.005	0.009
CA	-	-	< 0.001	< 0.001	< 0.001	0.001	0.002
CB	-	-	< 0.001	< 0.001	0.001	0.001	0.001
CC	-	-	< 0.001	< 0.001	< 0.001	0.001	0.001
CD	-	-	< 0.001	< 0.001	< 0.001	< 0.001	< 0.001
CE	-	-	< 0.001	< 0.001	< 0.001	0.001	0.001
CF	-	-	< 0.001	< 0.001	< 0.001	0.001	0.001
CG	-	-	< 0.001	< 0.001	< 0.001	< 0.001	0.001
CH	-	-	< 0.001	< 0.001	< 0.001	0.001	0.001
CI	-	-	< 0.001	< 0.001	< 0.001	< 0.001	< 0.001
CJ	-	-	< 0.001	0.001	0.007	0.001	0.009
CK	-	-	< 0.001	< 0.001	0.002	0.001	0.004
CL	-	-	< 0.001	< 0.001	< 0.001	0.002	0.003
CM	-	-	< 0.001	< 0.001	< 0.001	0.001	0.002
CN	-	-	< 0.001	< 0.001	< 0.001	< 0.001	< 0.001
CO	-	-	< 0.001	< 0.001	< 0.001	< 0.001	0.001

### Source contributions to ambient VOC concentrations at receptor sites

**Fig. 5:**
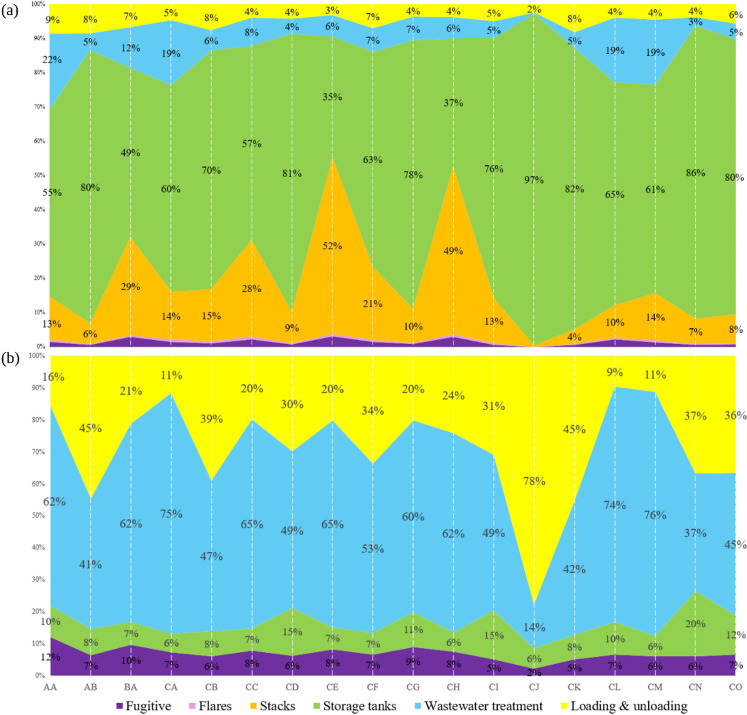
Source contribution to predicted annual concentrations of (**a**) benzene and. (**b**) 1,3-butadiene at receptor sites in 2022.

Source contribution analysis demonstrated distinct source signatures for benzene and 1,3-butadiene across receptor locations (Fig. 5). Ambient benzene concentrations were overwhelmingly dominated by storage tank emissions at most receptors, contributing between 81% and 97% at receptors CJ, CN, CK, and CD (Fig. 5a). Combustion-related sources provided secondary contributions, accounting for up to 52% at selected receptors influenced by nearby stacks, particularly at receptors in close proximity to PTC1, which operates the largest combustion stack within the estate and contributes 30.36% of facility-wide benzene emissions, resulting in elevated stack contributions at geometrically favored receptor locations. Contributions from wastewater treatment systems ranged from negligible to 22%, with the highest contribution observed at receptor AA. Flare contributions remained consistently below 2% at all receptors. In contrast, ambient 1,3-butadiene concentrations were governed primarily by wastewater treatment emissions, contributing 74–76% at receptors CM, CA, and CL (Fig. 5b). Loading and unloading operations served as secondary contributors, particularly at receptors located near material handling areas. Fugitive emissions contributed a relatively small fraction of total impacts, typically accounting for only 2–12% of overall concentrations, indicating that current leak detection and repair programs were generally effective. Nevertheless, sustained performance depends on regular inspection and preventive maintenance. The results further demonstrate a clear pollutant-specific hierarchy of emission sources, with benzene concentrations driven primarily by storage tank releases, whereas ambient levels of 1,3-butadiene were predominantly influenced by wastewater treatment operations. Consequently, air quality improvement within the industrial estate is most effectively achieved through source-targeted mitigation measures focusing on these dominant emission pathways.

### Decision-support analysis for emission control prioritization

The prioritization of benzene and 1,3-butadiene emission control strategies integrated atmospheric impact assessment with economic feasibility analysis. Control options were evaluated using cost-effectiveness indicators, NPV, and IRR over a 20-year project lifetime at a 10% discount rate. Economic benefits represent cost savings relative to baseline operating conditions, considering capital investment costs only, consistent with preliminary economic screening and corporate investment appraisal frameworks^55^^56^^57^,,. All monetary values are presented in Thai Baht (THB), with U.S. dollar (USD) equivalents based on a 2025 average exchange rate of 1 USD = 33 THB^69^.

#### Benzene emission control prioritization

Source characterization identified storage tanks as the dominant source of benzene emissions within the industrial estate, with releases strongly concentrated at a single vertical fixed-roof tank, establishing a clear priority for control. Six management scenarios were evaluated for benzene emission control, all targeting storage tanks as the primary source. BZ-S1 represents the baseline condition with poor physical integrity, while BZ-S2 reflects business-as-usual practice with routine maintenance. BZ-S3 and BZ-S4 convert tanks to internal floating roof configurations with vapor-mounted and liquid-mounted primary seals, respectively, while BZ-S5 upgrades to domed external floating roof tanks. BZ-S6 takes an alternative approach by installing activated carbon adsorption vapor recovery units (Table S14). Routine maintenance under BAU achieved a moderate emission reduction of approximately 24%, with corresponding decreases in receptor-level concentrations (Table S15). However, cost-effectiveness remained relatively low (Table S16), providing a benchmark for evaluating advanced control measures.

All proposed control measures achieved substantial benzene emission reductions of approximately 95 to 98% relative to BZ-S2 (Table S17), with receptor-level concentration decreases showing similar spatial patterns across scenarios and most locations exhibiting reductions of 40 to 70%. All four mitigation scenarios demonstrated markedly better cost-effectiveness than the business-as-usual baseline, with emission-based costs 34 to 50% lower and concentration-based costs 31 to 45% lower than BZ-S2 (Table 5). Among the evaluated options, BZ-S6 delivered the strongest overall performance by combining a 95% emission reduction with the lowest capital investment of approximately 15 million THB (USD 0.45 million). Structural modification under BZ-S3 required a moderate investment of about 23 million THB (USD 0.70 million) and achieved comparable emission and concentration reductions, while further performance gains from more advanced configurations required substantially higher investment with only marginal additional benefit.

**Table 5:** Cost-effectiveness of proposed benzene control measures. Note: * indicates values averaged across 18 receptor sites; exchange rate = 1 USD = 33 THB (2025 average).

Measures	Cost of investment (M THB)	Cost per unit of emission reduction		Cost per unit of ambient concentration reduction	
		Cost (THB/kg/y)	% Reduction of total emissions	Cost (M THB/0.1 µg/m^3^/y)	% Reduction in ambient concentrations*
BZ-S3	23.00 (USD 0.70 M)	4,476.70 (USD 136)	95.56	86.61 (USD 2.62 M)	52.28
BZ-S4	24.00 (USD 0.73 M)	4,626.32 (USD 140)	96.49	89.47 (USD 2.71 M)	52.81
BZ-S5	28.00 (USD 0.85 M)	5,289.57 (USD 160)	98.46	102.31 (USD 3.10 M)	53.88
BZ-S6	15.00 (USD 0.45 M)	2,936.85 (USD 89)	95.00	56.80 (USD 1.72 M)	51.98

Economic viability was assessed using NPV and IRR over a 20 year project lifetime with a 10% discount rate, using cost savings relative to the BZ-S2 baseline as benefits. Among the evaluated options, BZ-S6 generated the highest annual cost savings of approximately 15.1 million THB per year (USD 0.46 million per year), substantially exceeding other scenarios. Three options yielded positive NPVs, with BZ-S6 showing the strongest economic performance, achieving an NPV of 113.39 million THB (USD 3.44 million) and an IRR of 100.53% (Table 6). BZ-S3 and BZ-S4 also demonstrated economic feasibility, while BZ-S5 showed negative NPV and an IRR below the investment threshold despite achieving the highest emission reduction. Overall, BZ-S6 emerged as the economically optimal option, combining strong cost-effectiveness, high NPV, and IRR with the lowest capital investment, while BZ-S3 represented a robust alternative with comparable environmental performance at lower investment than higher-tier structural modifications.

**Table 6:** Economic evaluation of benzene emission control measures. Note: * indicates cost savings per kg reduced compared to BZ-S2 baseline (5,889.28 THB/kg/y; USD 178/kg/y); ** indicates annual cost savings, calculated as cost savings per unit multiplied by annual emission reduction. Discount rate: 10%; project lifetime: 20 years; exchange rate: 1 USD = 33 THB (2025 average).

Measures	Capital (M THB)	Annual reduction (kg/y)	Emission cost-effectiveness (THB/kg/y)	Cost savings* (THB/kg/y)	Annual savings** (M THB)	NPV (M THB)	IRR (%)
BZ-S3	23.00 (USD 0.70 M)	5,137.71	4,477 (USD 136)	1,412.58 (USD 43)	7.26 (USD 0.22 M)	38.80 (USD 1.18 M)	31.42
BZ-S4	24.00 (USD 0.73 M)	5,187.71	4,626 (USD 140)	1,262.96 (USD 38)	6.55 (USD 0.20 M)	31.79 (USD 0.96 M)	27.07
BZ-S5	28.00 (USD 0.85 M)	5,293.43	5,290 (USD 160)	599.71 (USD 18)	3.17 (USD 96 K)	−0.98 (USD − 30 K)	9.49
BZ-S6	15.00 (USD 0.45 M)	5,107.51	2,937 (USD 89)	2,952.43 (USD 89)	15.08 (USD 0.46 M)	113.38 (USD 3.44 M)	100.53

#### 1,3-Butadiene emission control prioritization

Analysis of emission sources showed that 1,3-butadiene releases were dominated by wastewater treatment operations, contributing roughly 63% of total emissions and originating mainly from a small number of units, with waste drop pipe 2 as the most influential source. To address this, six management scenarios were evaluated for 1,3-butadiene emission control, all targeting wastewater treatment systems. BD-S1 represents the baseline condition with uncovered open sumps, while BD-S2 reflects business-as-usual practice with covered equalization tanks. The core intervention across the remaining scenarios is the hard pipe with no headspace modification, applied to waste drop pipe unit 2 alone in BD-S3 or to both units 1 and 2 in BD-S4. BD-S5 and BD-S6 extend this by combining the hard pipe modification with aeration tank enclosure, targeting unit 2 only or both units, respectively (Table S18). Under BAU conditions, enclosing equalization tanks resulted in an emission reduction of approximately 25%, while corresponding decreases in modeled concentrations at receptor sites remained limited (Table S19). Consistent with this outcome, cost-effectiveness indicators under BAU exhibited comparatively high costs per unit concentration reduction (Table S20), highlighting the limited impact of diffuse wastewater sources on ambient levels and establishing a reference point for evaluating more advanced control options.

The ‘hard pipe with no headspace’ modification is a targeted engineering control that eliminates the primary volatilization pathway from wastewater drop pipes. Under the existing configuration (BD-S2), wastewater containing dissolved 1,3-butadiene is discharged through open drop pipes that maintain a gas-phase headspace, creating an air-liquid interface that allows continuous vapor escape driven by the concentration gradient between the dissolved and gas phases. The hard pipe modification replaces this with a sealed, fully enclosed piping system that connects the discharge point directly to the receiving treatment unit, eliminating the headspace and reducing the gas-liquid interface area to near zero. This approach is consistent with the closed-vent system and closed-drain principles recommended by U.S. EPA for controlling VOC emissions from wastewater systems at petroleum refineries^36^^70^,. Since waste drop pipe 2 accounted for approximately 98% of total wastewater-related 1,3-butadiene emissions under BAU conditions (Table S13), targeting this single unit (BD-S3) achieved a 97.89% reduction in total wastewater 1,3-butadiene emissions at a capital investment of only 0.20 M THB, representing a highly cost-effective intervention.

Across all four control strategies, emission reductions relative to BD-S2 were consistently high, ranging from 97.89% to 98.89%, while differences in receptor-level concentration reductions remained modest and spatially variable (Table S21). Although environmental performance was broadly comparable, economic performance varied substantially among options (Table 7). The simplest configuration, BD-S3, emerged as the most efficient choice, delivering nearly 98% emission abatement and more than 50% average concentration reduction with minimal capital input, which translated into the lowest cost-effectiveness indicators. By contrast, progressively more complex systems from BD-S4 to BD-S6 provided only limited incremental improvements in emission control at disproportionately higher costs, even though BD-S6 achieved the maximum overall reduction. Importantly, all mitigation scenarios performed markedly better than the business-as-usual case, lowering emission-based and concentration-based costs by approximately 86–92% and 89–92%, respectively, and thereby demonstrating a substantially improved conversion of emission reductions into ambient air quality benefits.

**Table 7:** Cost-effectiveness of proposed 1,3-butadiene control measures. Note: * indicates values averaged across 18 receptor sites; exchange rate = 1 USD = 33 THB (2025 average).

Measures	Cost of investment (M THB)	Cost per unit of emission reduction		Cost per unit of ambient concentration reduction	
		Cost (THB/kg/y)	% Reduction of total emissions	Cost (M THB/0.1 µg/m^3^/y)	% Reduction in ambient concentrations*
BD-S3	0.20 (USD 6 K)	244.47 (USD 7.41)	97.89	11.51 (USD 0.35 M)	52.99
BD-S4	0.40 (USD 12 K)	487.32 (USD 14.77)	98.22	22.86 (USD 0.69 M)	53.35
BD-S5	1.20 (USD 36 K)	1,456.76 (USD 44.14)	98.57	68.47 (USD 2.07 M)	53.43
BD-S6	1.40 (USD 42 K)	1,693.96 (USD 51.33)	98.89	79.38 (USD 2.41 M)	53.77

A 20 year investment appraisal was conducted using NPV and IRR at a 10% discount rate, with economic benefits defined as cost savings relative to the BD-S2 reference case (2,135.31 THB/kg/year; USD 65/kg/y). All control options were economically viable, yielding positive NPVs and IRRs well above the hurdle rate (Table 8). Among them, BD-S3 consistently outperformed the other scenarios, generating the highest annual cost savings and achieving the largest NPV at 13.00 M THB (USD 0.39 M) while requiring the smallest capital investment. More capital intensive options delivered progressively lower economic returns, despite comparable emission reduction performance. Based on both NPV and IRR, the overall economic ordering placed BD-S3 as the most favorable option, followed by BD-S4, BD-S5, and BD-S6, highlighting the advantage of targeted hard pipe modifications over more complex control systems.

**Table 8:** Economic evaluation of 1,3-butadiene emission control measures. Note: * indicates cost savings per kg reduced compared to BD-S2 baseline (2,135.31 THB/kg/y; USD 65/kg/y); ** indicates annual cost savings, calculated as cost savings per unit multiplied by annual emission reduction. Discount rate: 10%; project lifetime: 20 years; exchange rate: 1 USD = 33 THB (2025 average).

Measures	Capital (M THB)	Annual reduction (kg/y)	Emission cost-effectiveness (THB/kg/y)	Cost savings* (THB/kg/y)	Annual savings** (M THB)	NPV (M THB)	IRR (%)
BD-S3	0.2 (USD 6 K)	818.11	244 (USD 7.39)	1,890.84 (USD 57)	1.55 (USD 47 K)	13.00 (USD 0.39 M)	773
BD-S4	0.4 (USD 12 K)	820.82	487 (USD 14.76)	1,647.99 (USD 50)	1.35 (USD 41 K)	11.10 (USD 0.34 M)	338
BD-S5	1.2 (USD 36 K)	823.75	1,457 (USD 44.15)	678.55 (USD 21)	0.56 (USD 17 K)	3.60 (USD 0.11 M)	46.6
BD-S6	1.4 (USD 42 K)	826.46	1,694 (USD 51.33)	441.35 (USD 13)	0.36 (USD 11 K)	1.70 (USD 52 K)	25.8

#### Sensitivity analysis

To assess the robustness of the economic conclusions, sensitivity analysis was conducted on the economically viable options identified in Sect. 3.4.1 and 3.4.2. For benzene, BZ-S6 and BZ-S3 were selected as the two highest-ranked options based on NPV and cost-effectiveness performance, while BZ-S4 was excluded due to performance comparable to BZ-S3 at higher capital cost, and BZ-S5 was excluded due to its negative NPV under baseline conditions. For 1,3-butadiene, BD-S3 and BD-S4 were retained as the two most favorable options, while BD-S5 and BD-S6 were excluded as they provided only marginal emission reductions at disproportionately higher capital requirements. The discount rate was evaluated at 5%, 10% (base case), and 15% to reflect the range of capital costs applicable to industrial investments in developing economies^52^^[,53^.

**Table 9:** Sensitivity of NPV to discount rate variation for prioritized control options. Note: exchange rate = 1 USD = 33 THB (2025 average).

Scenario	Capital (M THB)	NPV at 5% (M THB)	NPV at 10% (M THB)	NPV at 15% (M THB)
BZ-S6	15.00 (USD 0.45 M)	172.93 (USD 5.24 M)	113.38 (USD 3.44 M)	79.39 (USD 2.41 M)
BZ-S3	23.00 (USD 0.70 M)	67.48 (USD 2.04 M)	38.81 (USD 1.18 M)	22.44 (USD 0.68 M)
BD-S3	0.20 (USD 6 K)	19.12 (USD 0.58 M)	13.00 (USD 0.39 M)	9.50 (USD 0.29 M)
BD-S4	0.40 (USD 12 K)	16.42 (USD 0.50 M)	11.09 (USD 0.34 M)	8.05 (USD 0.24 M)

All prioritized control options maintained positive NPV values across the full range of discount rates tested (Table 9), demonstrating that the economic conclusions are robust to discount rate assumptions. BZ-S6 retained the highest NPV among benzene options at every discount rate, ranging from 172.93 M THB (USD 5.24 M) at 5% to 79.39 M THB (USD 2.41 M) at 15%, while BZ-S3 also remained positive across all scenarios. For 1,3-butadiene, BD-S3 consistently outperformed BD-S4 at all discount rates tested. The ranking of prioritized options remained unchanged under all discount rate assumptions.

To evaluate the potential impact of O&M costs, a supplementary analysis was conducted assuming annual O&M expenditures of 5%, 10%, and 15% of capital investment, consistent with ranges reported for industrial emission control equipment^55^^46^,.

**Table 10:** Sensitivity of NPV to O&M cost assumptions (discount rate: 10%). Note: exchange rate = 1 USD = 33 THB (2025 average).

Scenario	Capital (M THB)	0% O&M (M THB)	5% O&M (M THB)	10% O&M (M THB)	15% O&M (M THB)
BZ-S6	15.00 (USD 0.45 M)	113.38 (USD 3.44 M)	107.00 (USD 3.24 M)	100.61 (USD 3.05 M)	94.23 (USD 2.86 M)
BZ-S3	23.00 (USD 0.70 M)	38.81 (USD 1.18 M)	29.02 (USD 0.88 M)	19.23 (USD 0.58 M)	9.44 (USD 0.29 M)
BD-S3	0.20 (USD 6 K)	13.00 (USD 0.39 M)	12.91 (USD 0.39 M)	12.83 (USD 0.39 M)	12.74 (USD 0.39 M)
BD-S4	0.40 (USD 12 K)	11.09 (USD 0.34 M)	10.92 (USD 0.33 M)	10.75 (USD 0.33 M)	10.58 (USD 0.32 M)

Even under the most conservative O&M assumption (15% of capital annually), all prioritized options retained strongly positive NPV values and the overall ranking remained unchanged (Table 10). BZ-S6 showed the highest resilience, with NPV decreasing from 113.38 M THB (USD 3.44 M) to 94.23 M THB (USD 2.86 M), representing only a 17% reduction. BZ-S3 exhibited greater sensitivity due to its higher capital base, though its NPV remained positive at 9.44 M THB (USD 0.29 M) under the 15% O&M scenario. BD-S3 and BD-S4 were minimally affected by O&M assumptions owing to their low capital requirements, with NPV reductions of less than 0.30 M THB across the full range. These results confirm that the capital-only economic conclusions reported in the main analysis are conservative and robust.

## Conclusions

This study presents an integrated framework that combines emission quantification, atmospheric dispersion modeling, source apportionment, and economic evaluation to support prioritization of VOC control strategies in a petroleum and petrochemical industrial estate in eastern Thailand. Focusing on benzene and 1,3-butadiene, the analysis identified dominant emission sources and demonstrated that sources exerting the greatest influence on ambient concentrations are not necessarily those with the highest emission mass, highlighting the importance of source–receptor relationships in exposure-oriented management.

Modeling results confirmed compliance with national ambient air quality standards, while source apportionment revealed that storage tanks dominated benzene contributions at key receptors, whereas wastewater treatment systems governed 1,3-butadiene exposure. Decision-support analysis integrating cost-effectiveness and investment appraisal metrics showed that lower-capital, source-specific interventions consistently delivered superior environmental and economic performance compared with more capital-intensive options. Emission reductions exceeding 95% were achieved with favorable cost-effectiveness ratios and strong economic returns, demonstrating that substantial air quality benefits can be realized without proportionally large capital investments.

This study is based on a single calendar year (2022) at one industrial estate. While multi-year data would broaden temporal coverage, the use of full-year operational and meteorological records ensures that seasonal variations are represented, consistent with U.S. EPA Appendix W regulatory guidance. The AERMOD inert tracer assumption is well justified for benzene given its atmospheric lifetime of approximately 9 days^3^, and remains appropriate for 1,3-butadiene at the near-field distances examined in this study, where transport times are short relative to its photochemical half-life^44^. Emission estimates carry uncertainties of approximately ± 20% for direct measurements and up to ± 50% for model-based calculations; uncertainty was not formally propagated across source categories but was treated as bounding values applied consistently with U.S. EPA guidance. These ranges are characteristic of standard EPA estimation protocols and do not affect the relative ranking of source contributions or control option prioritization, as the dominant contributions of storage tanks to benzene (81–97% at key receptors) and wastewater treatment to 1,3-butadiene (74–76%) are sufficiently large to be robust against plausible uncertainty bounds. The economic evaluation focuses on capital costs to enable consistent comparison across options, and sensitivity analysis incorporating O&M assumptions of up to 15% of capital annually confirmed that all prioritized options retained positive NPV values with unchanged rankings.

Despite these constraints, several findings are transferable to other petroleum and petrochemical contexts. The source dominance patterns observed in this study are consistent with multiple independent studies conducted at different facilities and in different countries^10^^[,13^^[,14^^[,61^, suggesting that these patterns reflect inherent process characteristics rather than site-specific anomalies. The integrated assessment framework is methodologically transferable and can be adapted to other industrial complexes by substituting site-specific emission data, meteorological inputs, and local cost parameters.

Overall, the proposed framework offers a transferable and evidence-based approach for prioritizing industrial emission control measures by linking emission dominance, ambient concentration response, and economic feasibility. This approach is particularly relevant for petrochemical complexes in resource-constrained settings, where efficient allocation of mitigation investments is essential to protect ambient air quality and public health.

## Supplementary Information

Below is the link to the electronic supplementary material.

Supplementary material 1 (DOCX 893.0 kb)

## Data Availability

The data are available from the corresponding author upon reasonable request.
