# Supplementary material for: Linking VOC assessment and cost-effectiveness for emission management in petroleum and petrochemical industrial estate
Source: Sci Rep. 2026 Apr 22;16:18734. doi: 10.1038/s41598-026-49628-3 (PMC13272899; doi:10.1038/s41598-026-49628-3)
Supplement: Supplementary file 1 — Supplementary material 1 (DOCX 893.0 kb) [file 41598_2026_49628_MOESM1_ESM.docx]

**Supplementary Materials:**

**Linking VOC Assessment and Cost-Effectiveness for Emission Management in Petroleum and Petrochemical Industrial Estate**

**Table 1.** Discrete receptor locations and other information.

| **Code** | **Receptors** | **UTM Coordinates (m)** | | **Distances from boundary (km)** |
| --- | --- | --- | --- | --- |
|  |  | **X** | **Y** |  |
| Air quality monitoring station around the IRPC Industrial Estate | | | | |
| AA | Wat Pluak Ket | 751369 | 1400708 | 0.22 |
| AB | Nong Chok Health Promoting Hospital | 750373 | 1402722 | 1.20 |
| Volatile organic compounds monitoring station | | | | |
| BA | IRPC Technology College | 751698 | 1401496 | 0.65 |
| Community areas and sensitive areas | | | | |
| CA | Sanam Pao Community | 754691 | 1399720 | 2.54 |
| CB | Pak Nam 2 Community | 751273 | 1404420 | 1.26 |
| CC | Prison Community | 755143 | 1401450 | 2.07 |
| CD | Village No. 4 Ban Don | 747678 | 1403710 | 4.15 |
| CE | Shopping Mall Community | 754780 | 1402870 | 1.77 |
| CF | Village No. 2 Ban Noen Chan | 752063 | 1405130 | 2.00 |
| CG | Village No. 3 Ban Nong Phaya | 745384 | 1400770 | 4.14 |
| CH | Village No. 7 Ban Nong Bua | 754602 | 1404060 | 1.85 |
| CI | Chai Krapom Community | 748731 | 1405330 | 3.67 |
| CJ | Wat Noen Phuttha | 749423 | 1400784 | 0.17 |
| CK | Ban Nong Chok School | 750141 | 1402333 | 1.44 |
| CL | Maha Sura Singhanat Camp | 752315 | 1398883 | 0.68 |
| CM | Wat Taphong Nok | 754715 | 1399460 | 2.71 |
| CN | Triwitthaya School | 747437 | 1402161 | 4.16 |
| CO | Ban Pak Khlong Municipality School | 746735 | 1400960 | 2.99 |

**Table S2.** Leak rate and screening value correlations for fugitive equipment for petroleum refinery plant.

| **Equipment type** | **Default zero emission rate (kg/hour/item)** | **Pegged emission rates (kg/hour/item)** | | **Correlations^*^**  **(kg/hour/item)** |
| --- | --- | --- | --- | --- |
|  |  | **10,000 ppmv** | **100,000 ppmv** |  |
| Connector | 7.50 × 10^-6^ | 0.028 | 0.030 | = 1.51 × 10^-6^ (SV)^0.735^ |
| Flange | 3.10 × 10^-7^ | 0.085 | 0.084 | = 4.41 × 10^-6^ (SV)^0.703^ |
| Open-ended line | 2.00 × 10^-6^ | 0.030 | 0.079 | = 2.16 × 10^-6^ (SV)^0.704^ |
| Pump | 2.40 × 10^-5^ | 0.074 | 0.160 | = 4.82 × 10^-5^ (SV)^0.610^ |
| Valve | 7.80 × 10^-6^ | 0.064 | 0.140 | = 2.28 × 10^-6^ (SV)^0.746^ |
| Other | 4.00 × 10^-6^ | 0.073 | 0.110 | = 1.32 × 10^-5^ (SV)^0.589^ |

*: SV is the net screening value (SV, ppmv) measured by the monitoring device.

**Table S3.** Leak rate and screening value correlations for fugitive equipment for petrochemical industry.

| **Equipment type** | **Default zero emission rate (kg/hour/item)** | **Pegged emission rates (kg/hour/item)** | | **Correlations***  **(kg/hour/item)** |
| --- | --- | --- | --- | --- |
|  |  | **10,000 ppmv** | **100,000 ppmv** |  |
| Gas/vapor valve | 6.60 × 10^-7^ | 0.024 | 0.110 | = 1.87 × 10^-6^ (SV)^0.873^ |
| Light liquid valve | 4.90 × 10^-7^ | 0.036 | 0.150 | = 6.41 × 10^-6^ (SV)^0.797^ |
| Pump | 7.50 × 10^-6^ | 0.140 | 0.620 | = 1.90 × 10^-5^ (SV)^0.824^ |
| Compressor | 7.50 × 10^-6^ | 0.140 | 0.620 | = 1.90 × 10^-5^ (SV)^0.824^ |
| Pressure relief valve | 7.50 × 10^-6^ | 0.140 | 0.620 | = 1.90 × 10^-5^ (SV)^0.824^ |
| Agitator | 7.50 × 10^-6^ | 0.140 | 0.620 | = 1.90 × 10^-5^ (SV)^0.824^ |
| Connector/flange | 6.10 × 10^-7^ | 0.044 | 0.220 | = 3.05 × 10^-6^ (SV)^0.885^ |

*: SV is the net screening value (SV, ppmv) measured by the monitoring device.

**Table S4.** Saturation (S) factors for calculating petroleum liquid loading losses.

| **Cargo carrier** | **Mode of operation** | **S factor** |
| --- | --- | --- |
| Tank tracks | Submerged loading of a clean cargo tank | 0.50 |
| Rail tank cars | Submerged loading: dedicated normal service | 0.60 |
|  | Submerged loading: dedicated vapor balance service | 1.00 |
|  | Splash loading of a clean cargo tank | 1.45 |
|  | Splash loading: dedicated normal service | 1.45 |
|  | Splash loading: dedicated vapor balance service | 1.00 |
| Marine vessels | Submerged loading: ships | 0.20 |
|  | Submerged loading: barges | 0.50 |

**Table S5.** Physical parameters and emission rates of fugitive source.

| **ID** | **UTM Coordinates (m)** | | **Release height (m)** | **Emission rate (g/s/m^2^)** | |
| --- | --- | --- | --- | --- | --- |
|  | **X** | **Y** |  | **Benzene** | **1,3-Butadiaene** |
| PTC1_FUGI | 752629.13 | 1402097.84 | 1.00 | 2.36E-07 | - |
|  | 752708.56 | 1402135.08 |  |  |  |
|  | 752787.71 | 1401960.80 |  |  |  |
|  | 752702.04 | 1401928.39 |  |  |  |
| PTC2_FUGI | 750028.20 | 1399607.33 | 1.00 | 3.54E-08 | - |
|  | 750172.05 | 1399550.82 |  |  |  |
|  | 750135.64 | 1399389.91 |  |  |  |
|  | 749962.40 | 1399451.92 |  |  |  |
| PTL1_FUGI | 750304.22 | 1400485.41 | 1.00 | 2.34E-09 | - |
|  | 750277.03 | 1400438.68 |  |  |  |
|  | 750387.27 | 1400396.60 |  |  |  |
|  | 750409.92 | 1400453.36 |  |  |  |
| PTL2_FUGI | 750375.79 | 1400835.43 | 1.00 | 2.62E-10 | - |
|  | 750537.18 | 1400773.77 |  |  |  |
|  | 750340.76 | 1400272.57 |  |  |  |
|  | 750217.66 | 1400318.96 |  |  |  |
| PTC5_FUGI | 750323.02 | 1400165.39 | 1.00 | 5.98E-09 | 2.14E-09 |
|  | 750501.06 | 1400100.67 |  |  |  |
|  | 750440.28 | 1399919.18 |  |  |  |
|  | 750242.42 | 1400000.11 |  |  |  |
| PTL3_FUGI | 752392.49 | 1401958.17 | 1.00 | 3.75E-10 | - |
|  | 752473.83 | 1401782.03 |  |  |  |
|  | 752173.65 | 1401710.97 |  |  |  |
|  | 752108.13 | 1401856.25 |  |  |  |
| PTL5_FUGI | 752029.48 | 1402782.01 | 1.00 | 2.01E-10 | - |
|  | 752376.69 | 1402341.85 |  |  |  |
|  | 752045.60 | 1402216.39 |  |  |  |
|  | 751813.90 | 1402694.54 |  |  |  |
| PTC3_FUGI | 750637.43 | 1400164.40 | 1.00 | - | 3.06E-06 |
|  | 750631.25 | 1400137.68 |  |  |  |
|  | 750602.05 | 1400146.72 |  |  |  |
|  | 750611.18 | 1400171.14 |  |  |  |
| PTC4_FUGI | 750573.65 | 1399892.47 | 1.00 | - | 1.32E-07 |
|  | 750665.38 | 1399854.88 |  |  |  |
|  | 750645.72 | 1399803.79 |  |  |  |
|  | 750553.75 | 1399843.82 |  |  |  |

**Table S6.** Physical parameters and emission rates of flare.

| **ID** | **Flare** | | **Exit gas** | | **UTM Coordinates (m)** | | **Emission rate (g/s)** | |
| --- | --- | --- | --- | --- | --- | --- | --- | --- |
|  | **Height  (m)** | **Diameter  (m)** | **Velocity  (m/s)** | **Temp.  (K)** | **X** | **Y** | **Benzene** | **1,3-Butadiaene** |
| PTC1_FLARE | 120.00 | 0.71 | 3.00 | 1273.2 | 752720.00 | 1402087.00 | 0.02400 | - |
| PTC2_FLARE | 144.00 | 0.88 | 3.00 | 1273.2 | 752721.00 | 1402078.00 | 0.01200 | - |
| PTC4_FLARE | 120.00 | 1.07 | 3.00 | 1273.2 | 752723.00 | 1402074.00 | 0.01200 | - |
| PTL1_FLARE | 120.00 | 0.71 | 3.00 | 1273.2 | 752709.00 | 1401940.00 | 0.01800 | - |
| PTL2_FLARE | 150.00 | 0.71 | 3.00 | 1273.2 | 752716.00 | 1401925.00 | 0.05400 | - |
| PTC5_FLARE | 150.00 | 0.71 | 3.00 | 1273.2 | 750085.00 | 1399579.00 | 0.00001 | - |
| PTL4_FLARE | 150.00 | 0.71 | 3.00 | 1273.2 | 750095.00 | 1399574.00 | 0.00001 | - |
| PTL5_FLARE | 150.00 | 1.52 | 3.00 | 1273.2 | 750105.00 | 1399570.00 | 0.00000 | - |

**Table S7.** Physical parameters and emission rates of stationary combustion unit.

| **ID** | **Stack** | | **Exit gas** | | **UTM Coordinates (m)** | | **Emission rate (g/s)** | |
| --- | --- | --- | --- | --- | --- | --- | --- | --- |
|  | **Height  (m)** | **Diameter  (m)** | **Velocity  (m/s)** | **Temp.  (K)** | **X** | **Y** | **Benzene** | **1,3-Butadiaene** |
| PTC1_02B001 | 40.00 | 1.82 | 10.60 | 579.15 | 752720.00 | 1402087.00 | 0.02400 | - |
| PTC1_02B002 | 40.00 | 1.05 | 6.20 | 559.15 | 752721.00 | 1402078.00 | 0.01200 | - |
| PTC1_02B003 | 18.00 | 0.45 | 4.20 | 549.15 | 752723.00 | 1402074.00 | 0.01200 | - |
| PTC1_03B001 | 40.00 | 1.54 | 8.60 | 458.15 | 752709.00 | 1401940.00 | 0.01800 | - |
| PTC1_03B002 | 40.00 | 2.76 | 6.80 | 461.15 | 752716.00 | 1401925.00 | 0.05400 | - |
| PTC2_01B001 | 19.80 | 1.25 | 7.47 | 465.15 | 750085.00 | 1399579.00 | 0.00001 | - |
| PTC2_01B002 | 19.80 | 1.25 | 7.78 | 468.15 | 750095.00 | 1399574.00 | 0.00001 | - |
| PTC2_03B001 | 16.80 | 1.00 | 7.22 | 530.15 | 750105.00 | 1399570.00 | 0.00000 | - |
| PTC4_B7801 | 65.00 | 3.15 | 5.10 | 401.15 | 750270.00 | 1399618.00 | 0.00010 | - |
| PTC4_F0101 | 42.00 | 2.00 | 15.60 | 393.15 | 750550.00 | 1399796.00 | 0.00005 | - |
| PTC4_F0201 | 42.00 | 2.00 | 15.60 | 393.15 | 750565.00 | 1399791.00 | 0.00005 | - |
| PTC4_F0301 | 42.00 | 2.00 | 15.60 | 393.15 | 750579.00 | 1399785.00 | 0.00005 | - |
| PTC4_F0401 | 42.00 | 2.00 | 15.60 | 393.15 | 750593.00 | 1399779.00 | 0.00005 | - |
| PTC4_F0501 | 42.00 | 2.00 | 15.60 | 393.15 | 750605.00 | 1399774.00 | 0.00005 | - |
| PTC4_F0601 | 42.00 | 1.90 | 16.00 | 393.15 | 750619.00 | 1399770.00 | 0.00005 | - |
| PTL1_ADU1A | 50.76 | 1.98 | 5.79 | 562.15 | 750739.00 | 1400149.00 | 0.00002 | - |
| PTL1_ADU1B | 53.38 | 2.17 | 6.43 | 513.15 | 750732.00 | 1400152.00 | 0.00003 | - |
| PTL1_15B001 | 26.46 | 1.52 | 5.49 | 578.15 | 750622.00 | 1399949.00 | 0.00001 | - |
| PTL1_10B001 | 32.96 | 1.22 | 4.94 | 593.15 | 750782.00 | 1400216.00 | 0.00000 | - |
| PTL1_10B002 | 25.43 | 1.42 | 5.00 | 608.15 | 750804.00 | 1400230.00 | 0.00000 | - |
| PTL1_12B001 | 38.80 | 1.10 | 6.00 | 583.15 | 750798.00 | 1400256.00 | 0.00001 | - |
| PTL1_12B002 | 38.80 | 1.10 | 6.00 | 583.15 | 750798.00 | 1400256.00 | 0.00001 | - |
| PTL1_12B003 | 38.80 | 1.10 | 6.00 | 583.15 | 750798.00 | 1400256.00 | 0.00001 | - |
| PTL1_12B004 | 38.80 | 2.75 | 10.86 | 583.15 | 750798.00 | 1400256.00 | 0.00001 | - |
| PTL1_12B005 | 22.80 | 1.15 | 4.62 | 498.15 | 750789.00 | 1400244.00 | 0.00000 | - |
| PTL2_ADU2A | 57.50 | 3.00 | 10.00 | 623.15 | 750375.00 | 1400798.00 | 0.00006 | - |
| PTL2_ADU2B | 57.50 | 2.70 | 7.70 | 624.15 | 750387.00 | 1400795.00 | 0.00006 | - |
| PTL2_SRU | 60.00 | 2.20 | 7.90 | 574.15 | 750138.00 | 1399880.00 | 0.00000 | - |
| PTC5_30B001 | 28.00 | 1.17 | 6.56 | 573.15 | 750318.00 | 1399986.00 | 0.00001 | - |
| PTC5_30B002 | 40.00 | 1.65 | 7.17 | 543.15 | 750327.00 | 1399970.00 | 0.00002 | - |
| PTC5_31B002 | 30.00 | 1.52 | 9.61 | 517.15 | 750356.00 | 1400100.00 | 0.00001 | - |
| PTC5_33B002 | 23.00 | 0.88 | 3.46 | 613.15 | 750403.00 | 1399950.00 | 0.00000 | - |
| PTL4_25B001 | 20.00 | 1.00 | 9.10 | 460.15 | 752163.00 | 1401849.00 | 0.00000 | - |
| PTL4_24B001 | 34.74 | 2.13 | 5.80 | 483.15 | 752142.00 | 1401817.00 | 0.00002 | - |
| PTL4_22B001 | 45.00 | 2.27 | 8.60 | 539.15 | 752179.00 | 1401735.00 | 0.00001 | - |
| PTL4_22B002 | 38.25 | 1.28 | 6.10 | 593.15 | 752180.00 | 1401724.00 | 0.00002 | - |
| PTL4_21B001 | 34.59 | 1.80 | 8.50 | 503.15 | 752130.00 | 1401837.00 | 0.00002 | - |
| PTL5_51Z002 | 60.00 | 2.60 | 9.40 | 433.15 | 752086.00 | 1402276.00 | 0.00020 | - |
| PTL5_52B001 | 60.00 | 1.60 | 10.50 | 452.15 | 752025.00 | 1402383.00 | 0.00001 | - |
| PTL5_53B101 | 60.00 | 2.11 | 4.40 | 453.15 | 752095.00 | 1402410.00 | 0.00002 | - |
| PTL5_54B001 | 60.00 | 0.90 | 10.70 | 481.15 | 752347.00 | 1402351.00 | 0.00000 | - |
| PTL5_73Z401 | 60.00 | 1.20 | 12.70 | 513.15 | 752011.00 | 1402735.00 | 0.00000 | - |

**Table S8.** Physical parameters and emission rates of storage tank.

| **ID** | **Tank types** | **Chemical** | **Tank** | | **Exit gas** | | **UTM Coordinates (m)** | | **Emission rate (g/s)** | |
| --- | --- | --- | --- | --- | --- | --- | --- | --- | --- | --- |
|  |  |  | **Height** | **Diameter** | **Velocity** | **Temp.** | **X** | **Y** | **Benzene** | **1,3-Butadiaene** |
|  |  |  | **(m)** | **(m)** | **(m/s)** | **(K)** |  |  |  |  |
| PTC1_06T009 | IFR | BZ/TOL mix tank | 14.94 | 0.2 | 0.1 | 305.15 | 752795.38 | 1401831.71 | 0.00056 | - |
| PTC1_06T010 | IFR | BZ day tank | 17.09 | 0.2 | 0.1 | 305.15 | 752807.30 | 1401872.55 | 0.00236 | - |
| PTC2_06T011 | VFR | Raffinate Tank | 12.19 | 0.2 | 0.1 | 305.15 | 749999.32 | 1399517.32 | 0.00005 | - |
| PTC2_06T013A | VFR | Toluene Day Tank | 9.5 | 0.2 | 0.1 | 305.15 | 750112.36 | 1399479.58 | 0.00002 | - |
| PTC2_06T013B | VFR | Toluene Day Tank B | 9.5 | 0.2 | 0.1 | 305.15 | 750103.59 | 1399463.34 | 0.00002 | - |
| PTC2_06T015 | VFR | Ethyl Xylene Tank with mixxylene | 13 | 0.2 | 0.1 | 305.15 | 750083.85 | 1399446.23 | 0.00001 | - |
| PTC2_06T016 | VFR | Mix Xylene Tank | 16.3 | 0.2 | 0.1 | 305.15 | 750039.79 | 1399451.49 | 0.00009 | - |
| PTC2_06T018 | VFR | Toluene Tank | 17.7 | 0.2 | 0.1 | 305.15 | 750059.56 | 1399489.18 | 0.00013 | - |
| PTC2_06T001 | IFR | C7- Reformate Feed Tank | 11.6 | 0.2 | 0.1 | 305.15 | 750013.45 | 1399492.98 | 0.00007 | - |
| PTC2_06T002 | IFR | Reformate Extract Rundown Tank | 9.9 | 0.2 | 0.1 | 305.15 | 750013.45 | 1399492.98 | 0.00007 | - |
| PTC2_06T005 | IFR | Py_gas Feed Tank | 15.9 | 0.2 | 0.1 | 305.15 | 749985.60 | 1399483.55 | 0.00039 | - |
| PTC2_06T006 | IFR | Py_gas Extract Product Tank | 11.3 | 0.2 | 0.1 | 305.15 | 750005.87 | 1399477.53 | 0.00031 | - |
| PTC2_06T009 | IFR | Reformate Feed Tank | 14.9 | 0.2 | 0.1 | 305.15 | 750016.36 | 1399569.15 | 0.00003 | - |
| PTC2_06T0010 | IFR | Pygas Import | 17.1 | 0.2 | 0.1 | 305.15 | 750007.91 | 1399541.30 | 0.00073 | - |
| PTC2_06T0012A | IFR | Benzene Day Tank | 8.1 | 0.2 | 0.1 | 305.15 | 750094.40 | 1399481.74 | 0.00056 | - |
| PTC2_06T0012B | IFR | Benzene Day Tank | 8.1 | 0.2 | 0.1 | 305.15 | 750090.17 | 1399468.42 | 0.00039 | - |
| PTC2_06T0014A | IFR | BT Slop Day Tank | 6.7 | 0.2 | 0.1 | 305.15 | 750121.21 | 1399473.57 | 0.00001 | - |
| PTC2_06T0014B | IFR | SLOP DAY TANK | 9.4 | 0.2 | 0.1 | 305.15 | 750115.35 | 1399460.23 | 0.00000 | - |
| PTC2_06T0020 | IFR | Mix Xylene | 9.4 | 0.2 | 0.1 | 305.15 | 750077.19 | 1399424.81 | 0.00606 | - |
| PTC2_81T0001 | IFR | Bezene tank To EBSM | 14.6 | 0.2 | 0.1 | 305.15 | 750120.52 | 1399428.85 | 0.00291 | - |
| PTC4_D9503 | IFR | Pyrolysis Gasoline | 21.5 | 0.2 | 0.1 | 305.15 | 750369.04 | 1399674.50 | 0.00160 | - |
| PTC4_D9506 | IFR | C6-C8 Heartcut | 21.1 | 0.2 | 0.1 | 305.15 | 750411.62 | 1399646.09 | 0.00160 | - |
| PTL1_69T001 | EFR | CRUDE TANK | 16.8 | 0.2 | 0.1 | 305.15 | 749958.23 | 1400111.04 | 0.00024 | - |
| PTL1_69T002C | EFR | CRUDE OIL TANK | 14.4 | 0.2 | 0.1 | 305.15 | 749835.34 | 1400033.92 | 0.00016 | - |
| PTL1_69T003A | EFR | Light Naphtha tank | 11.9 | 0.2 | 0.1 | 305.15 | 749763.36 | 1400088.19 | 0.00104 | - |
| PTL1_69T003B | EFR | Light Naphtha tank | 11.9 | 0.2 | 0.1 | 305.15 | 749775.35 | 1400121.05 | 0.00107 | - |
| PTL1_69T003C | EFR | Light Naphtha tank | 11.9 | 0.2 | 0.1 | 305.15 | 749735.50 | 1400141.17 | 0.00077 | - |
| PTL1_69T003D | EFR | Light Naphtha tank | 11.9 | 0.2 | 0.1 | 305.15 | 749720.71 | 1400105.96 | 0.00104 | - |
| PTL1_69T004A | EFR | Heavy NAPHTHA TANK | 14.5 | 0.2 | 0.1 | 305.15 | 749814.55 | 1400210.71 | 0.00133 | - |
| PTL1_69T004B | EFR | Heavy NAPHTHA TANK | 14.5 | 0.2 | 0.1 | 305.15 | 749793.77 | 1400163.82 | 0.00086 | - |
| PTL1_69T004C | EFR | Heavy NAPHTHA TANK | 14.5 | 0.2 | 0.1 | 305.15 | 749750.91 | 1400181.48 | 0.00083 | - |
| PTL1_69T004D | EFR | Heavy NAPHTHA TANK | 14.4 | 0.2 | 0.1 | 305.15 | 749770.89 | 1400232.36 | 0.00127 | - |
| PTL1_69T005A | EFR | Full length NAPHTHA TANK | 14.5 | 0.2 | 0.1 | 305.15 | 749746.82 | 1400053.63 | 0.00087 | - |
| PTL1_69T005B | EFR | Full length NAPHTHA TANK | 14.5 | 0.2 | 0.1 | 305.15 | 749704.83 | 1400069.97 | 0.00086 | - |
| PTL1_69T011A | EFR | SLOP TANK | 12.1 | 0.2 | 0.1 | 305.15 | 749943.11 | 1400248.04 | 0.00018 | - |
| PTL1_69T011B | DFR | SLOP TANK | 12.1 | 0.2 | 0.1 | 305.15 | 749952.08 | 1400611.68 | 0.00001 | - |
| PTL1_69T022 | IFR | GASOLINE TANK | 14.5 | 0.2 | 0.1 | 305.15 | 749943.11 | 1400248.04 | 0.00084 | - |
| PTL1_69T023 | EFR | CRUDE TANK | 17 | 0.2 | 0.1 | 305.15 | 749926.36 | 1400016.03 | 0.00024 | - |
| PTL1_69T024A | IFR | GASOLINE TANK | 15.8 | 0.2 | 0.1 | 305.15 | 750039.36 | 1400289.72 | 0.00010 | - |
| PTL1_69T024B | IFR | GASOLINE TANK | 15.8 | 0.2 | 0.1 | 305.15 | 750019.83 | 1400298.07 | 0.00000 | - |
| PTL1_69T024C | IFR | GASOLINE TANK | 15.8 | 0.2 | 0.1 | 305.15 | 750059.10 | 1400281.93 | 0.00043 | - |
| PTL1_69T025A | IFR | GASOLINE TANK | 12.4 | 0.2 | 0.1 | 305.15 | 749959.10 | 1400283.93 | 0.00040 | - |
| PTL1_69T025B | IFR | GASOLINE TANK | 12.4 | 0.2 | 0.1 | 305.15 | 749970.23 | 1400315.79 | 0.00054 | - |
| PTL1_69T025C | IFR | GASOLINE TANK | 12.4 | 0.2 | 0.1 | 305.15 | 749942.83 | 1400330.27 | 0.00054 | - |
| PTL1_69T025D | IFR | GASOLINE TANK | 12.4 | 0.2 | 0.1 | 305.15 | 749930.61 | 1400298.40 | 0.00036 | - |
| PTL1_69T025E | IFR | GASOLINE TANK | 12.4 | 0.2 | 0.1 | 305.15 | 750044.08 | 1400482.01 | 0.00050 | - |
| PTL1_69T025F | IFR | GASOLINE TANK | 12.4 | 0.2 | 0.1 | 305.15 | 750058.58 | 1400513.90 | 0.00045 | - |
| PTL1_69T025G | IFR | Gasoline Tank | 12.5 | 0.2 | 0.1 | 305.15 | 750012.35 | 1400494.23 | 0.00073 | - |
| PTL1_69T025H | IFR | GASOLINE TANK | 12.5 | 0.2 | 0.1 | 305.15 | 750025.98 | 1400526.12 | 0.00067 | - |
| PTL1_69T027B | IFR | GASOLINE Tank | 14.3 | 0.2 | 0.1 | 305.15 | 749946.71 | 1400506.59 | 0.00034 | - |
| PTL1_69T050A | EFR | CRUDE TANK | 20.1 | 0.2 | 0.1 | 305.15 | 749713.34 | 1399955.60 | 0.00029 | - |
| PTL1_69T050B | EFR | CRUDE TANK | 20 | 0.2 | 0.1 | 305.15 | 749825.93 | 1399906.35 | 0.00029 | - |
| PTL1_69T050C | EFR | CRUDE OIL TANK | 20.1 | 0.2 | 0.1 | 305.15 | 749779.06 | 1399798.47 | 0.00029 | - |
| PTL1_69T050D | EFR | CRUDE OIL TANK | 20.1 | 0.2 | 0.1 | 305.15 | 749666.92 | 1399845.18 | 0.00030 | - |
| PTL1_69T061A | IFR | T-HN TANK | 11.9 | 0.2 | 0.1 | 305.15 | 749772.61 | 1400666.87 | 0.00005 | - |
| PTL1_69T061B | IFR | T-HN TANK | 11.9 | 0.2 | 0.1 | 305.15 | 749789.44 | 1400706.08 | 0.00005 | - |
| PTL1_69T066 | IFR | GASOLINE TANK | 12.5 | 0.2 | 0.1 | 305.15 | 749750.27 | 1400612.44 | 0.00075 | - |
| PTL1_69T088A | VFR | REFORMATE TANK | 14.6 | 0.2 | 0.1 | 305.15 | 749442.80 | 1400479.03 | 0.17048 | - |
| PTL2_69T011C | DFR | SLOP TANK | 12.2 | 0.2 | 0.1 | 305.15 | 749952.08 | 1400611.67 | 0.00000 | - |
| PTL2_69T011D | DFR | SLOP TANK | 12.1 | 0.2 | 0.1 | 305.15 | 749952.08 | 1400611.67 | 0.00019 | - |
| PTL2_69T050E | EFR | CRUDE TANK | 19.5 | 0.2 | 0.1 | 305.15 | 749400.39 | 1399950.08 | 0.00033 | - |
| PTL2_69T050F | EFR | CRUDE OIL TANK | 19.6 | 0.2 | 0.1 | 305.15 | 749515.92 | 1399899.86 | 0.00033 | - |
| PTL2_69T050G | EFR | CRUDE TANK | 19.5 | 0.2 | 0.1 | 305.15 | 749560.05 | 1400010.48 | 0.00030 | - |
| PTL2_69T050H | EFR | CRUDE TANK | 19.5 | 0.2 | 0.1 | 305.15 | 749605.27 | 1400121.44 | 0.00033 | - |
| PTL2_69T050I | EFR | CRUDE TANK | 19.5 | 0.2 | 0.1 | 305.15 | 749651.78 | 1400232.07 | 0.00034 | - |
| PTL2_69T080B | EFR | CRUDE TANK | 21 | 0.2 | 0.1 | 305.15 | 749670.50 | 1400686.55 | 0.00026 | - |
| PTL2_69T080C | EFR | CRUDE TANK | 19.6 | 0.2 | 0.1 | 305.15 | 749513.49 | 1400622.42 | 0.00019 | - |
| PTL2_69T080D | EFR | CRUDE TANK | 19.6 | 0.2 | 0.1 | 305.15 | 749560.33 | 1400732.95 | 0.00020 | - |
| PTC5_63T006A | IFR | Untreated Gasoline | 14 | 0.2 | 0.1 | 305.15 | 750413.99 | 1399506.33 | 0.00072 | - |
| PTC5_63T006B | IFR | Treated Gasoline | 14 | 0.2 | 0.1 | 305.15 | 750394.58 | 1399515.00 | 0.00074 | - |
| PTC5_63T006C | IFR | Treated Gasoline | 14 | 0.2 | 0.1 | 305.15 | 750386.36 | 1399490.66 | 0.00074 | - |
| PTL5_77T005B | IFR | Naphtha | 16.7 | 0.2 | 0.1 | 305.15 | 751596.74 | 1402782.28 | 0.00090 | - |
| PTL5_77T014 | IFR | Off spec Naphatha | 17.7 | 0.2 | 0.1 | 305.15 | 751683.77 | 1402757.70 | 0.00028 | - |
| PTL6_T95B | IFR | ULG91 | 8.9 | 0.2 | 0.1 | 305.15 | 750001.49 | 1400708.55 | 0.00055 | - |
| PTL6_T95C | IFR | ULG91 | 8.9 | 0.2 | 0.1 | 305.15 | 749993.50 | 1400736.31 | 0.00032 | - |
| PTL6_T95D | IFR | ULG91 | 8.9 | 0.2 | 0.1 | 305.15 | 750011.38 | 1400728.63 | 0.00032 | - |
| PTL6_T95E | IFR | ULG91 | 8.9 | 0.2 | 0.1 | 305.15 | 750011.60 | 1400683.10 | 0.00045 | - |
| PTL6_T95F | IFR | ULG91 | 8.9 | 0.2 | 0.1 | 305.15 | 750020.95 | 1400701.57 | 0.00045 | - |
| PTC3_04T002 | VFR | PBDE latex tank 200 m3 | 7.6 | 0.2 | 0.1 | 305.15 | 750661.24 | 1400162.03 | - | 0.00075 |
| PTC3_04T003 | VFR | PBDE latex tank 200 m3 | 7.6 | 0.2 | 0.1 | 305.15 | 750665.53 | 1400171.60 | - | 0.00161 |
| PTC3_04T005 | VFR | PBDE latex tank 200 m3 | 7.6 | 0.2 | 0.1 | 305.15 | 750666.51 | 1400146.53 | - | 0.00074 |

**Table S9.** Physical parameters and emission rates of wastewater treatment.

| **ID** | **UTM Coordinates (m)** | | **Release height (m)** | **Emission rate (g/s/m^2^)** | |
| --- | --- | --- | --- | --- | --- |
|  | **X** | **Y** |  | **Benzene** | **1,3-Butadiaene** |
| WWT | 750464.31 | 1399782.60 | 1.00 | 2.52E-06 | 2.01E-06 |
|  | 750425.74 | 1399731.79 |  |  |  |
|  | 750611.53 | 1399650.65 |  |  |  |
|  | 750636.44 | 1399722.25 |  |  |  |

**Table S10.** Physical parameters and emission rates of loading operation.

| **ID** | **UTM Coordinates (m)** | | **Release height (m)** | **Emission rate (g/s/m^2^)** | |
| --- | --- | --- | --- | --- | --- |
|  | **X** | **Y** |  | **Benzene** | **1,3-Butadiaene** |
| PTL6 | 749956.90 | 1400799.79 | 1.00 | 6.24E-06 | 3.12E-06 |
|  | 749992.82 | 1400781.84 |  |  |  |
|  | 749956.90 | 1400708.02 |  |  |  |
|  | 749922.99 | 1400723.98 |  |  |  |

**Table S11.** Summary of scenario analysis.

| **Situation** | **Scenario** | **Target source** | **Detailed** |
| --- | --- | --- | --- |
| **Benzene Management Scenarios** | | | |
| Absence of emission control measures | BZ-S1: Poor physical condition of storage tank | Storage tank | Storage tank exhibited deteriorated conditions in both the tank shell and roof structure |
| Current situation | BZ-S2: Business as usual (BAU) | Storage tank | Storage tank received comprehensive maintenance to restore good physical condition |
| Implementation of additional measures | BZ-S3: Converting to IFRT with vapor-mounted primary seal and rim-mounted secondary seal | Storage tank | Storage tank type will be changed to Internal Floating Roof Tank (IFRT) with the following specifications:  Tank Physical Characteristics:   - Self-Supporting Roof: No - Number of Support Columns: 7 - Effective Column Diameter: 1 ft - Internal Shell Condition: Light Rust - External Shell Color: White/White - External Shell Condition: Good - Roof Color: White/White - Roof Paint Condition: Good |
|  |  |  | Rim-Seal System:   - Primary Seal: Vapor-mounted - Secondary Seal: Rim-mounted |
|  |  |  | Deck Characteristics:   - Deck Type: Welded - Deck Fitting Category: Typical |
|  | BZ-S4: Converting to IFRT with liquid-mounted primary seal and rim-mounted secondary seal | Storage tank | Storage tank type will be changed to Internal Floating Roof Tank (IFRT) with the following specifications:  Tank Physical Characteristics:   - Self-Supporting Roof: No - Number of Support Columns: 7 - Effective Column Diameter: 1 ft - Internal Shell Condition: Light Rust - External Shell Color: White/White - External Shell Condition: Good - Roof Color: White/White - Roof Paint Condition: Good |
|  |  |  | Rim-Seal System:   - Primary Seal: Liquid-mounted - Secondary Seal: Rim-mounted |
|  |  |  | Deck Characteristics:   - Deck Type: Welded - Deck Fitting Category: Typical |
|  | BZ-S5: Converting to DEFRT | Storage tank | Storage tank type will be changed to Domed External Floating Roof Tank (DEFRT) with the following specifications:  Tank Physical Characteristics:   - Internal Shell Condition: Light Rust - Paint Color/Shade: White/White - Paint Condition: Good |
|  |  |  | Roof Characteristics:   - Roof Type: Pontoon - Roof Fitting Category: Typical |
|  |  |  | Tank Construction and Rim-Seal System:   - Tank Construction: Welded - Primary Seal: Vapor-mounted - Secondary Seal: Rim-mounted |
|  | BZ-S6: Installing activated carbon adsorption VRU system | Storage tank | Installing activated carbon adsorption VRU system in the storage tank |
| **1,3-Butadiene Management Scenarios** | | | |
| Absence of emission control measures | BD-S1: Uncovered open sump in WWT | Wastewater treatment unit | Open sump in wastewater treatment system was uncovered |
| Current situation | BD-S2: Business as usual (BAU) | Wastewater treatment unit | Equalization tanks were covered |
| Implementation of additional measures | BD-S3: Changing to hard pipe no head space in unit 2 only | Wastewater treatment unit | Waste drop from pipe in unit 2 only will be modified into a hard pipe with no headspace |
|  | BD-S4: Changing to hard pipe no head space in unit 1 and 2 | Wastewater treatment unit | Waste drop from pipe in unit 1 and 2 will be modified into a hard pipe with no headspace |
|  | BD-S5: Changing to hard pipe no head space in unit 2 only and enclosing aeration tank | Wastewater treatment unit | Waste drop from pipe in unit 2 only will be converted to a hard pipe with no headspace system and Aeration (09T206) in wastewater treatment unit 2 will be fully enclosed |
|  | BD-S6: Changing to hard pipe no head space in unit 1 and 2 and enclosing aeration tank | Wastewater treatment unit | Waste drop from pipe in unit 1 and 2 will be converted to a hard pipe with no headspace system and Aeration (09T206) in wastewater treatment unit 2 will be fully enclosed |

**Table S12.** Summary of benzene and 1,3-butadiene emissions from storage tanks in year 2022.

| **Plant** | **Tank ID** | **Tank types** | **Type of chemical stored** | **Emission rate (kg/year) and percent contribution (%)** | | | |
| --- | --- | --- | --- | --- | --- | --- | --- |
|  |  |  |  | **Benzene** | | **1,3-Butadiene** | |
|  |  |  |  | **Emission rate** | **%** | **Emission rate** | **%** |
| PTC1 | 06T009 | IFRT | BZ/TOL | 17.70 | 0.26 | 0.00 | 0.00 |
|  | 06T010 | IFRT | BZ | 74.50 | 1.10 | 0.00 | 0.00 |
| PTC2 | 06T011 | VFRT | Raffinate | 1.42 | 0.02 | 0.00 | 0.00 |
|  | 06T013A | VFRT | Toluene | 0.50 | 0.01 | 0.00 | 0.00 |
|  | 06T013B | VFRT | Toluene | 0.51 | 0.01 | 0.00 | 0.00 |
|  | 06T015 | VFRT | Ethyl Xylene | 0.27 | 0.00 | 0.00 | 0.00 |
|  | 06T016 | VFRT | Mix Xylene | 2.91 | 0.04 | 0.00 | 0.00 |
|  | 06T018 | VFRT | Toluene | 3.95 | 0.06 | 0.00 | 0.00 |
|  | 06T001 | IFRT | C7- Reformate | 2.27 | 0.03 | 0.00 | 0.00 |
|  | 06T002 | IFRT | Reformate Extract | 2.10 | 0.03 | 0.00 | 0.00 |
|  | 06T005 | IFRT | Pygas | 12.40 | 0.18 | 0.00 | 0.00 |
|  | 06T006 | IFRT | Pygas Extract Product | 9.83 | 0.14 | 0.00 | 0.00 |
|  | 06T009 | IFRT | Reformate | 1.08 | 0.02 | 0.00 | 0.00 |
|  | 06T010 | IFRT | Pygas Import | 23.04 | 0.34 | 0.00 | 0.00 |
|  | 06T012A | IFRT | Benzene | 17.65 | 0.26 | 0.00 | 0.00 |
|  | 06T012B | IFRT | Benzene | 12.44 | 0.18 | 0.00 | 0.00 |
|  | 06T014A | IFRT | BT Slop | 0.17 | 0.00 | 0.00 | 0.00 |
|  | 06T014B | IFRT | SLOP | 0.07 | 0.00 | 0.00 | 0.00 |
|  | 06T020 | IFRT | Mix Xylene | 191.12 | 2.82 | 0.00 | 0.00 |
|  | 81T0001 | IFRT | Benzene | 91.90 | 1.35 | 0.00 | 0.00 |
| PTC3 | 04T002 | VFRT | PBDE latex | 0.00 | 0.00 | 23.62 | 24.11 |
|  | 04T003 | VFRT | PBDE latex | 0.00 | 0.00 | 50.86 | 51.91 |
|  | 04T005 | VFRT | PBDE latex | 0.00 | 0.00 | 23.49 | 23.98 |
| PTC4 | D9503 | IFRT | Pyrolysis Gasoline | 50.52 | 0.74 | 0.00 | 0.00 |
|  | D9506 | IFRT | C6-C8 Heart cut | 50.52 | 0.74 | 0.00 | 0.00 |
| PTL1 | 69T001 | EFRT | Crude | 7.63 | 0.11 | 0.00 | 0.00 |
|  | 69T002C | EFRT | Crude Oil | 4.94 | 0.07 | 0.00 | 0.00 |
|  | 69T003A | EFRT | Light Naphtha | 32.76 | 0.48 | 0.00 | 0.00 |
|  | 69T003B | EFRT | Light Naphtha | 33.69 | 0.50 | 0.00 | 0.00 |
|  | 69T003C | EFRT | Light Naphtha | 24.32 | 0.36 | 0.00 | 0.00 |
|  | 69T003D | EFRT | Light Naphtha | 32.93 | 0.49 | 0.00 | 0.00 |
|  | 69T004A | EFRT | Heavy Naphtha | 42.03 | 0.62 | 0.00 | 0.00 |
|  | 69T004B | EFRT | Heavy Naphtha | 27.21 | 0.40 | 0.00 | 0.00 |
|  | 69T004C | EFRT | Heavy Naphtha | 26.12 | 0.39 | 0.00 | 0.00 |
|  | 69T004D | EFRT | Heavy Naphtha | 40.16 | 0.59 | 0.00 | 0.00 |
|  | 69T005A | EFRT | Full length Naphtha | 27.56 | 0.41 | 0.00 | 0.00 |
|  | 69T005B | EFRT | Full length Naphtha | 27.15 | 0.40 | 0.00 | 0.00 |
|  | 69T011A | EFRT | SLOP | 5.80 | 0.09 | 0.00 | 0.00 |
|  | 69T011B | DFRT | SLOP | 0.43 | 0.01 | 0.00 | 0.00 |
|  | 69T022 | IFRT | Gasoline | 26.48 | 0.39 | 0.00 | 0.00 |
|  | 69T023 | EFRT | Crude | 7.67 | 0.11 | 0.00 | 0.00 |
|  | 69T024A | IFRT | Gasoline | 3.29 | 0.05 | 0.00 | 0.00 |
|  | 69T024B | IFRT | Gasoline | 0.12 | 0.00 | 0.00 | 0.00 |
|  | 69T024C | IFRT | Gasoline | 13.55 | 0.20 | 0.00 | 0.00 |
|  | 69T025A | IFRT | Gasoline | 12.60 | 0.19 | 0.00 | 0.00 |
|  | 69T025B | IFRT | Gasoline | 17.17 | 0.25 | 0.00 | 0.00 |
|  | 69T025C | IFRT | Gasoline | 17.19 | 0.25 | 0.00 | 0.00 |
|  | 69T025D | IFRT | Gasoline | 11.41 | 0.17 | 0.00 | 0.00 |
|  | 69T025E | IFRT | Gasoline | 15.63 | 0.23 | 0.00 | 0.00 |
|  | 69T025F | IFRT | Gasoline | 14.33 | 0.21 | 0.00 | 0.00 |
|  | 69T025G | IFRT | Gasoline | 23.03 | 0.34 | 0.00 | 0.00 |
|  | 69T025H | IFRT | Gasoline | 21.12 | 0.31 | 0.00 | 0.00 |
|  | 69T027B | IFRT | Gasoline | 10.76 | 0.16 | 0.00 | 0.00 |
|  | 69T050A | EFRT | Crude | 9.08 | 0.13 | 0.00 | 0.00 |
|  | 69T050B | EFRT | Crude | 9.09 | 0.13 | 0.00 | 0.00 |
|  | 69T050C | EFRT | Crude Oil | 9.15 | 0.13 | 0.00 | 0.00 |
|  | 69T050D | EFRT | Crude Oil | 9.39 | 0.14 | 0.00 | 0.00 |
|  | 69T061A | IFRT | T-HN | 1.68 | 0.02 | 0.00 | 0.00 |
|  | 69T061B | IFRT | T-HN | 1.63 | 0.02 | 0.00 | 0.00 |
|  | 69T066 | IFRT | Gasoline | 23.61 | 0.35 | 0.00 | 0.00 |
|  | 69T088A | VFRT | Reformate | 5,376.32 | 79.26 | 0.00 | 0.00 |
| PTL2 | 69T011C | DFRT | SLOP | 0.02 | 0.00 | 0.00 | 0.00 |
|  | 69T011D | DFRT | SLOP | 5.98 | 0.09 | 0.00 | 0.00 |
|  | 69T050E | EFRT | Crude | 10.27 | 0.15 | 0.00 | 0.00 |
|  | 69T050F | EFRT | Crude Oil | 10.46 | 0.15 | 0.00 | 0.00 |
|  | 69T050G | EFRT | Crude | 9.34 | 0.14 | 0.00 | 0.00 |
|  | 69T050H | EFRT | Crude | 10.36 | 0.15 | 0.00 | 0.00 |
|  | 69T050I | EFRT | Crude | 10.70 | 0.16 | 0.00 | 0.00 |
|  | 69T080B | EFRT | Crude | 8.07 | 0.12 | 0.00 | 0.00 |
|  | 69T080C | EFRT | Crude | 6.14 | 0.09 | 0.00 | 0.00 |
|  | 69T080D | EFRT | Crude | 6.37 | 0.09 | 0.00 | 0.00 |
| PTC5 | 63T006A | IFRT | Untreated Gasoline | 22.62 | 0.33 | 0.00 | 0.00 |
|  | 63T006B | IFRT | Treated Gasoline | 23.28 | 0.34 | 0.00 | 0.00 |
|  | 63T006C | IFRT | Treated Gasoline | 23.20 | 0.34 | 0.00 | 0.00 |
| PTL5 | 77T005B | IFRT | Naphtha | 28.35 | 0.42 | 0.00 | 0.00 |
|  | 77T014 | IFRT | Off spec Naphtha | 8.76 | 0.13 | 0.00 | 0.00 |
| PTL6 | T95B | IFRT | ULG91 | 17.39 | 0.26 | 0.00 | 0.00 |
|  | T95C | IFRT | ULG91 | 10.04 | 0.15 | 0.00 | 0.00 |
|  | T95D | IFRT | ULG91 | 10.04 | 0.15 | 0.00 | 0.00 |
|  | T95E | IFRT | ULG91 | 14.10 | 0.21 | 0.00 | 0.00 |
|  | T95F | IFRT | ULG91 | 14.09 | 0.21 | 0.00 | 0.00 |
| **Total emission** | | | | **6,783.50** | **100.00** | **97.97** | **100.00** |

**Table S13.** Benzene and 1,3-butadiene emissions and percentage of contributions from individual units in wastewater treatment plants in 2022.

| **Unit** | **Emission rate (kg/year) and percent contribution (%)** | | | |
| --- | --- | --- | --- | --- |
|  | **Benzene** | | **1,3-Butadiene** | |
|  | **Emission rate** | **%** | **Emission rate** | **%** |
| Waste drop from pipe1 | 2.64E-01 | 0.03 | 9.27E+00 | 1.11 |
| WWT Sump | 2.81E-02 | 0.00 | 5.20E-01 | 0.06 |
| DAF1 WWT1 | 4.45E-02 | 0.00 | 8.77E-01 | 0.10 |
| DAF2 WWT1 | 6.87E-02 | 0.01 | 1.28E+00 | 0.15 |
| Equalization Tank | 3.07E-19 | 0.00 | 0.00E+00 | 0.00 |
| Aeration Tank | 6.91E-07 | 0.00 | 0.00E+00 | 0.00 |
| Sedimentation Tank A | 8.77E-10 | 0.00 | 0.00E+00 | 0.00 |
| Sedimentation Tank B | 6.69E-10 | 0.00 | 0.00E+00 | 0.00 |
| Equalization Tank (09T102) | 3.07E-09 | 0.00 | 0.00E+00 | 0.00 |
| Aeration (09T106) | 4.89E-13 | 0.00 | 0.00E+00 | 0.00 |
| Sedimentation Tank 4/1 | 9.08E-16 | 0.00 | 0.00E+00 | 0.00 |
| UF Package | 3.91E-15 | 0.00 | 0.00E+00 | 0.00 |
| Waste drop from pipe2 | 1.04E+03 | 99.91 | 8.22E+02 | 98.37 |
| Permeate Tank 4/1 (09T124) | 0.00E+00 | 0.00 | 0.00E+00 | 0.00 |
| Treated Oily Water Basin (D8710) | 0.00E+00 | 0.00 | 0.00E+00 | 0.00 |
| CPI Splitter Box (87T015) | 0.00E+00 | 0.00 | 0.00E+00 | 0.00 |
| CPI Tank E (Y8714E) | 0.00E+00 | 0.00 | 0.00E+00 | 0.00 |
| CPI Tank F (Y8714F) | 0.00E+00 | 0.00 | 0.00E+00 | 0.00 |
| CPI Tank G (Y8714G) | 0.00E+00 | 0.00 | 0.00E+00 | 0.00 |
| Equalization Tank (D8712) | 9.81E-14 | 0.00 | 6.75E-14 | 0.00 |
| Equalization Tank (09T202) | 4.35E-15 | 0.00 | 5.77E-15 | 0.00 |
| Aeration (09T206) | 7.92E-01 | 0.08 | 1.65E+00 | 0.20 |
| Sedimentation Tank 4/2 | 7.57E-05 | 0.00 | 1.31E-04 | 0.00 |
| UF Membrane Unit | 4.67E-04 | 0.00 | 7.66E-04 | 0.00 |
| Permeate Tank 4/2 (09T224) | 2.51E-07 | 0.00 | 3.66E-06 | 0.00 |
| Pond2 | 3.25E-08 | 0.00 | 0.00E+00 | 0.00 |
| **Total emission** | **1,047.02** | **100.00** | **835.70** | **100.00** |

**Table S14.** Detailed information of scenario analysis for benzene emission management.

| **Scenario** | **Scenario details** | **Costs of investment**  **(M THB)** |
| --- | --- | --- |
| **BZ-S1:** Poor physical condition of storage tank | Storage tank exhibited deteriorated conditions in both the tank shell and roof structure | - |
| **BZ-S2:** Business as usual (BAU) | Storage tank received comprehensive maintenance to restore good physical condition | 10.00 |
| **BZ-S3:** Converting to IFRT with vapor-mounted primary seal and rim-mounted secondary seal | Storage tank type will be changed to Internal Floating Roof Tank (IFRT). | 23.00 |
| **BZ-S4:** Converting to IFRT with liquid-mounted primary seal and rim-mounted secondary seal | Storage tank type will be changed to Internal Floating Roof Tank (IFRT). | 24.00 |
| **BZ-S5:** Converting to DEFRT | Storage tank type will be changed to Domed External Floating Roof Tank (DEFRT). | 28.00 |
| **BZ-S6:** Installing activated carbon adsorption VRU system | Installing activated carbon adsorption VRU on the existing VFRT without tank modification | 15.00 |

**Table S15.** Comparison of benzene emission rates, annual concentrations at receptors, and percent reductions between baseline (BZ-S1) and good physical condition (BZ-S2) scenarios.

| **Receptors** | **benzene emission rate (kg/y)** | | **benzene annual concentration (µg/m^3^)** | | **BZ-S2** | |
| --- | --- | --- | --- | --- | --- | --- |
|  | **BZ-S1** | **BZ-S2** | **BZ-S1** | **BZ-S2** | **% Emission reduction** | **% Concentration reduction** |
| AA | 7,074.33 | 5,376.32 | 0.0804 | 0.0712 | 23.95 | 11.42 |
| AB |  |  | 0.0356 | 0.0292 |  | 17.78 |
| BA |  |  | 0.0609 | 0.0545 |  | 10.51 |
| CA |  |  | 0.0100 | 0.0087 |  | 12.46 |
| CB |  |  | 0.0155 | 0.0131 |  | 15.30 |
| CC |  |  | 0.0147 | 0.0129 |  | 12.49 |
| CD |  |  | 0.0081 | 0.0067 |  | 17.39 |
| CE |  |  | 0.0203 | 0.0188 |  | 7.54 |
| CF |  |  | 0.0132 | 0.0114 |  | 13.87 |
| CG |  |  | 0.0073 | 0.0061 |  | 16.99 |
| CH |  |  | 0.0181 | 0.0166 |  | 8.32 |
| CI |  |  | 0.0060 | 0.0051 |  | 16.06 |
| CJ |  |  | 0.7511 | 0.5793 |  | 22.88 |
| CK |  |  | 0.0480 | 0.0391 |  | 18.42 |
| CL |  |  | 0.0160 | 0.0141 |  | 12.29 |
| CM |  |  | 0.0092 | 0.0080 |  | 12.42 |
| CN |  |  | 0.0117 | 0.0095 |  | 18.35 |
| CO |  |  | 0.0114 | 0.0095 |  | 17.25 |

**Table S16.** Cost effectiveness analysis of benzene in terms of business as usual.

| **Measures** | **Cost of investment  (M THB)** | **Cost per unit of emission reduction** | | **Cost per unit of ambient concentration reduction** | |
| --- | --- | --- | --- | --- | --- |
|  |  | **Cost (THB/kg/y)** | **% Reduction of total emissions** | **Cost**  **(M THB/0.1 µg/m^3^/y)** | **% Reduction in ambient concentrations^*^** |
| BZ-S2: BAU  Storage tank was good physical condition | 10.00 | 5,889.28 | 23.95 | 80.46 | 14.54 |

^*^ Ambient concentrations are the average value of annual concentrations of eighteen receptor sites.

**Table S17.** Percentage of reduction of benzene emission and concentration of mitigation measures .

| **Receptors** | **BZ-S3** | | **BZ-S4** | | **BZ-S5** | | **BZ-S6** | |
| --- | --- | --- | --- | --- | --- | --- | --- | --- |
|  | **% Emission reduction** | **% Concentration reduction** | **% Emission reduction** | **% Concentration reduction** | **% Emission reduction** | **% Concentration reduction** | **% Emission reduction** | **% Concentration reduction** |
| AA | 95.56 | 38.99 | 96.49 | 39.36 | 98.46 | 40.17 | 95.00 | 38.76 |
| AB |  | 65.38 |  | 66.03 |  | 67.36 |  | 65.00 |
| BA |  | 35.55 |  | 35.90 |  | 36.62 |  | 35.33 |
| CA |  | 43.05 |  | 43.51 |  | 44.32 |  | 42.82 |
| CB |  | 54.73 |  | 55.26 |  | 56.40 |  | 54.42 |
| CC |  | 43.13 |  | 43.60 |  | 44.45 |  | 42.90 |
| CD |  | 63.76 |  | 64.51 |  | 65.71 |  | 63.46 |
| CE |  | 24.69 |  | 24.91 |  | 25.44 |  | 24.53 |
| CF |  | 48.77 |  | 49.21 |  | 50.26 |  | 48.50 |
| CG |  | 61.88 |  | 62.54 |  | 63.86 |  | 61.55 |
| CH |  | 27.42 |  | 27.72 |  | 28.26 |  | 27.30 |
| CI |  | 58.38 |  | 58.97 |  | 60.16 |  | 57.99 |
| CJ |  | 89.74 |  | 90.61 |  | 92.46 |  | 89.21 |
| CK |  | 68.27 |  | 68.93 |  | 70.34 |  | 67.88 |
| CL |  | 42.39 |  | 42.82 |  | 43.74 |  | 42.18 |
| CM |  | 43.28 |  | 43.78 |  | 44.65 |  | 43.03 |
| CN |  | 68.28 |  | 68.91 |  | 70.27 |  | 67.86 |
| CO |  | 63.28 |  | 63.92 |  | 65.29 |  | 62.96 |

**Table S18.** Detailed information of scenario analysis for 1,3-butadiene emission management.

| **Scenario** | **Scenario details** | **Costs of investment**  **(M THB)** |
| --- | --- | --- |
| **BD-S1:** Uncovered open sump | Open sump in wastewater treatment system was uncovered | - |
| **BD-S2:** BAU | Equalization tanks were covered | 0.60 |
| **BD-S3:** Hard pipe with no head space unit 2 | Waste drop from pipe in unit 2 only will be modified into a hard pipe with no headspace | 0.20 |
| **BD-S4:** Hard pipe with no head space unit 1 and 2 | Waste drop from pipe in unit 1 and 2 will be modified into a hard pipe with no headspace | 0.40 |
| **BD-S5:** Hard pipe with no head space unit 2 and Aeration enclosing | Waste drop from pipe in unit 2 only will be converted to a hard pipe with no headspace system and Aeration (09T206) in wastewater treatment unit 2 will be fully enclosed | 1.20 |
| **BD-S6:** Hard pipe with no head space unit 1, 2 and aeration enclosing | Waste drop from pipe in unit 1 and 2 will be converted to a hard pipe with no headspace system and Aeration (09T206) in wastewater treatment unit 2 will be fully enclosed | 1.40 |

**Table S19.** Comparison of 1,3-butadiene emission rates, annual concentrations at receptors, and percent reductions between baseline (BD-S1) and covered eq basin (BD-S2) scenarios.

| **Receptors** | **1,3-butadiene emission rate (kg/y)** | | **1,3-butadiene annual concentration (µg/m^3^)** | | **BD-S2** | |
| --- | --- | --- | --- | --- | --- | --- |
|  | **BD-S1** | **BD-S2** | **BD-S1** | **BD-S2** | **% Emission reduction** | **% Concentration reduction** |
| AA | 1,116.69 | 835.70 | 0.0241 | 0.0199 | 25.16 | 17.32 |
| AB |  |  | 0.0031 | 0.0027 |  | 11.90 |
| BA |  |  | 0.0104 | 0.0086 |  | 17.20 |
| CA |  |  | 0.0022 | 0.0018 |  | 20.45 |
| CB |  |  | 0.0015 | 0.0013 |  | 14.00 |
| CC |  |  | 0.0016 | 0.0013 |  | 18.06 |
| CD |  |  | 0.0006 | 0.0005 |  | 14.55 |
| CE |  |  | 0.0018 | 0.0014 |  | 18.18 |
| CF |  |  | 0.0014 | 0.0012 |  | 15.60 |
| CG |  |  | 0.0007 | 0.0006 |  | 16.67 |
| CH |  |  | 0.0016 | 0.0013 |  | 17.39 |
| CI |  |  | 0.0005 | 0.0004 |  | 13.33 |
| CJ |  |  | 0.0094 | 0.0090 |  | 4.45 |
| CK |  |  | 0.0040 | 0.0035 |  | 12.25 |
| CL |  |  | 0.0036 | 0.0029 |  | 19.89 |
| CM |  |  | 0.0020 | 0.0016 |  | 20.30 |
| CN |  |  | 0.0006 | 0.0005 |  | 10.91 |
| CO |  |  | 0.0009 | 0.0007 |  | 12.94 |

**Table S20.** Cost effectiveness analysis of 1,3-butadiene in terms of business as usual.

| **Measures** | **Cost of investment  (M THB)** | **Cost per unit of emission reduction** | | **Cost per unit of ambient concentration reduction** | |
| --- | --- | --- | --- | --- | --- |
|  |  | **Cost (THB/kg/y)** | **% Reduction of total emissions** | **Cost**  **(M THB/0.1 µg/m^3^/y)** | **% Reduction in ambient concentrations^*^** |
| BD-S2: BAU  Equalization tanks  were covered | 0.60 | 2,135.31 | 25.16 | 102,564,102.56 | 15.30 |

^*^ ambient concentrations are the average value of annual concentrations of eighteen receptor sites.

**Table S21.** Percentage of reduction of 1,3-butadiene emission and concentration of mitigation measures.

| **Receptors** | **BD-S3** | | | **BD-S4** | | **BD-S5** | | **BD-S6** | |
| --- | --- | --- | --- | --- | --- | --- | --- | --- | --- |
|  | **% Emission reduction** | **% Concentration reduction** | **% Emission reduction** | | **% Concentration reduction** | **% Emission reduction** | **% Concentration reduction** | **% Emission reduction** | **% Concentration reduction** |
| AA | 97.89 | 61.16 | 98.22 | | 61.36 | 98.57 | 61.61 | 98.89 | 61.81 |
| AB |  | 39.78 |  |  | 40.15 |  | 40.15 |  | 40.51 |
| BA |  | 60.68 |  |  | 60.91 |  | 61.14 |  | 61.26 |
| CA |  | 73.71 |  |  | 73.71 |  | 74.29 |  | 74.29 |
| CB |  | 46.51 |  |  | 46.51 |  | 46.51 |  | 47.29 |
| CC |  | 63.78 |  |  | 63.78 |  | 63.78 |  | 64.57 |
| CD |  | 46.81 |  |  | 48.94 |  | 48.94 |  | 48.94 |
| CE |  | 62.50 |  |  | 63.19 |  | 63.19 |  | 63.19 |
| CF |  | 52.10 |  |  | 52.10 |  | 52.10 |  | 52.10 |
| CG |  | 58.18 |  |  | 60.00 |  | 60.00 |  | 60.00 |
| CH |  | 60.90 |  |  | 61.65 |  | 61.65 |  | 61.65 |
| CI |  | 46.15 |  |  | 46.15 |  | 46.15 |  | 48.72 |
| CJ |  | 13.30 |  |  | 13.30 |  | 13.41 |  | 13.41 |
| CK |  | 40.74 |  |  | 41.03 |  | 41.03 |  | 41.31 |
| CL |  | 72.38 |  |  | 72.38 |  | 72.73 |  | 73.08 |
| CM |  | 75.16 |  |  | 75.16 |  | 75.16 |  | 75.78 |
| CN |  | 36.73 |  |  | 36.73 |  | 36.73 |  | 36.73 |
| CO |  | 43.24 |  |  | 43.24 |  | 43.24 |  | 43.24 |


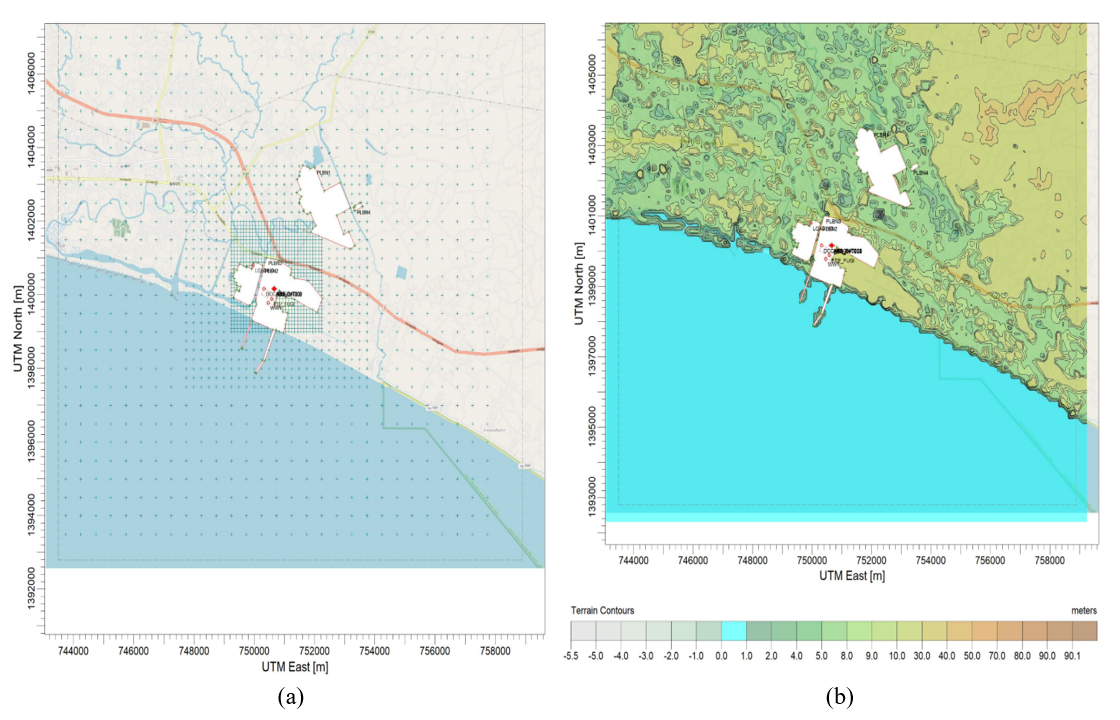


**Fig. S1.** (a) Study domain and (b) terrain elevations. Generated using AERMOD version 9.8.3 (U.S. EPA, https://www.epa.gov/scram/air-quality-dispersion-modeling-preferred-and-recommended-models).
